# T-Cell Engagers in Lung Cancer: A Comprehensive Literature Review from Tarlatamab Approval to Next-Generation Strategies

**DOI:** 10.3390/cancers18172725

**Published:** 2026-08-22

**Authors:** Adnan Saydawi, Sameh Madanieh, Stephanie L. Echeverria, Angad Gill, Sweta Modha, Beyan El Emin, Waqar Haider, Bsher Almaalouli, Mohamed Shanshal

**Affiliations:** 1 Department of Internal Medicine, McLaren Oakland Hospital, Michigan State University, Pontiac, MI 48342, USA; adnan.saydawi@mclaren.org; 2 Department of Internal Medicine, University of Central Florida, Lake Nona, FL 32827, USA; smaeh.madaneih@ucf.edu (S.M.); angad.gill@ucf.edu (A.G.); sweta.modha@ucf.edu (S.M.); beyan.elemin@ucf.edu (B.E.E.); waqar.haider@ucf.edu (W.H.); bsher.almaalouli@ucf.edu (B.A.); 3 Division of Hematology/Oncology, Vanderbilt University Medical Center, Nashville, TN 37232, USA

**Keywords:** T-cell engager, bispecific antibody, lung cancer, small cell lung cancer, non-small cell lung cancer, tarlatamab, DLL3, cytokine release syndrome, tumor microenvironment, immunotherapy

## Abstract

Background: Lung cancer remains the leading cause of cancer-related mortality worldwide, with five-year survival below 5% for metastatic small cell lung cancer (SCLC) and below 10% for metastatic non-small cell lung cancer (NSCLC). Immune checkpoint inhibitors have improved outcomes, but primary and acquired resistance, driven by tumor microenvironment immunosuppression, antigen heterogeneity, and T-cell exhaustion, leaves a substantial unmet need. T-cell engagers (TCEs), bispecific antibodies that redirect cytotoxic T-cells to tumor cells independent of MHC-I-restricted antigen presentation, offer a mechanistically distinct approach. Methods: We conducted a structured narrative review, without formal PRISMA methodology or meta-analytic pooling, of PubMed, Embase, and ClinicalTrials.gov through June 2026, supplemented by conference abstracts from ASCO, ESMO, AACR, and ATS, covering clinical, translational, and preclinical evidence for TCEs across established and emerging targets in thoracic malignancy. Results: Tarlatamab, a DLL3/CD3 bispecific TCE, received full FDA approval in November 2025 based on DeLLphi-304 data showing a median overall survival benefit of 13.6 versus 8.3 months over chemotherapy (HR 0.60; *p* < 0.001), establishing proof-of-concept for the TCE platform in lung cancer and NCCN Category 1 status in ES-SCLC. Beyond DLL3, an expanding pipeline of targets, including Claudin-18.2, TROP-2, FOLR1, CD70, and HER2, is under active TCE development; several of these antigens have independently validated tumor-selective expression through approved or late-stage antibody-drug conjugates (ADCs), providing target-level clinical de-risking for TCE development, though the two modalities have distinct requirements for antigen density and internalization that must be independently validated. Novel tri-specific constructs incorporating costimulatory domains and combination strategies with checkpoint inhibitors are in early clinical development. Conclusions: Tarlatamab approval validates the TCE platform in lung cancer, but overcoming TME-mediated resistance, antigen heterogeneity, and class-specific toxicities including cytokine release syndrome remains the central challenge. Rational TCE design, incorporating costimulatory signaling, antigen selection informed by parallel ADC validation data, and evidence-based combination strategies, offers the most credible path toward expanding this platform’s impact in metastatic lung cancer.

## Simple Summary

Lung cancer is the leading cause of cancer death worldwide, and patients whose disease has spread throughout the body have very few effective treatment options. A new class of drugs called T-cell engagers works by physically connecting the body’s own immune cells to cancer cells, directing the immune system to attack the tumor. One such drug, tarlatamab, recently received full approval from the U.S. Food and Drug Administration for patients with advanced small cell lung cancer that has returned after initial chemotherapy, after a large clinical trial showed it significantly extended survival compared to standard chemotherapy. This review summarizes the current evidence supporting tarlatamab, examines its safety profile, and explores a broad pipeline of similar drugs targeting different proteins on lung cancer cells. Understanding these emerging therapies and the challenges they face may help guide future research aimed at improving outcomes for patients with advanced lung cancer.

## 1. Introduction

According to global cancer statistics, lung cancer accounts for an estimated 2.5 million new cases and 1.8 million deaths annually [1]. Metastatic disease contributes disproportionately to this burden, accounting for approximately 60% of lung cancer deaths, and is characterized by advanced stage at diagnosis, high mortality, and resistance to conventional therapies [2]. Tumor resistance is driven by a combination of factors including tumor heterogeneity, systemic spread, and immunosuppressive microenvironments. The poor prognosis is reflected by five-year survival rates below 5% for metastatic SCLC and below 10% for metastatic NSCLC, highlighting a clear need for innovative treatment strategies to extend survival and improve quality of life [3].

Although immunotherapy significantly improves the outcome of patients with lung cancer, the effectiveness of these therapies is compromised by immune escape mechanisms that enable tumors to evade immune surveillance and develop resistance to treatment. Treatment resistance occurs via (1) failure of antigen recognition, (2) deficiency of antigen presentation, and (3) exhaustion of CD8+ T cells [4,5].

**Figure 1:**
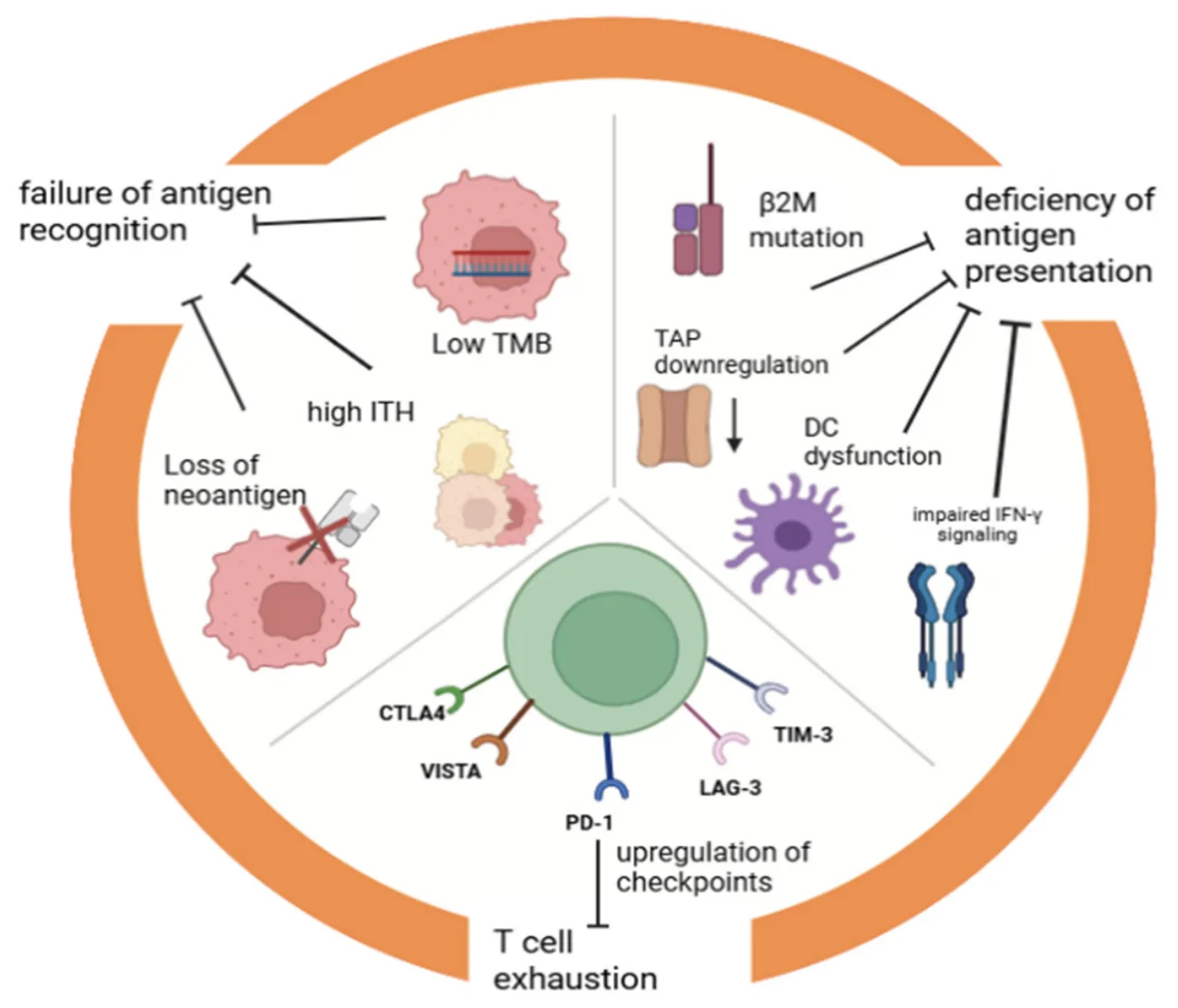
Mechanisms of immune checkpoint inhibitor resistance. TAP, transporters associated with neoantigen presentation; TMB, tumor mutation burden; ITH, intra-tumor heterogeneity; DC, dendritic cell; CTLA-4, cytotoxic T-lymphocyte antigen 4; VISTA, V-domain Ig suppressor of T cell activation; LAG-3, lymphocyte activation gene-3; PD-1, programmed cell death protein-1; TIM-3, T-cell immunoglobulin mucin-3. (Figure created with BioRender.com).

Among the resistance mechanisms depicted in Figure 1, MHC-I downregulation carries particular relevance to the rationale for TCE therapy. Because conventional cytotoxic T-lymphocyte responses and checkpoint inhibitor efficacy both depend on MHC-I-restricted antigen presentation, tumors that downregulate MHC-I, a common and well-documented immune escape strategy, become functionally invisible to these T-cell receptor-based mechanisms even when checkpoint blockade is otherwise successful in restoring T-cell function. TCEs directly circumvent this specific escape route: by physically linking a tumor-associated antigen to CD3 independent of the T-cell receptor and MHC-I complex, TCE-mediated cytotoxicity proceeds regardless of a tumor’s MHC-I expression status. This mechanistic independence is a central part of the rationale for pursuing TCEs specifically in settings where MHC-I downregulation contributes to immune evasion, discussed further in Section 3.

Cellular immunotherapies, including chimeric antigen receptor (CAR) T-cell therapy and tumor-infiltrating lymphocyte (TIL) therapy, are also being explored for metastatic lung malignancies [6,7,8,9,10]. CAR T-cell therapy is under active investigation in NSCLC and SCLC, though early clinical experience has raised concerns about severe off-target toxicity [11].

**Figure 2:**
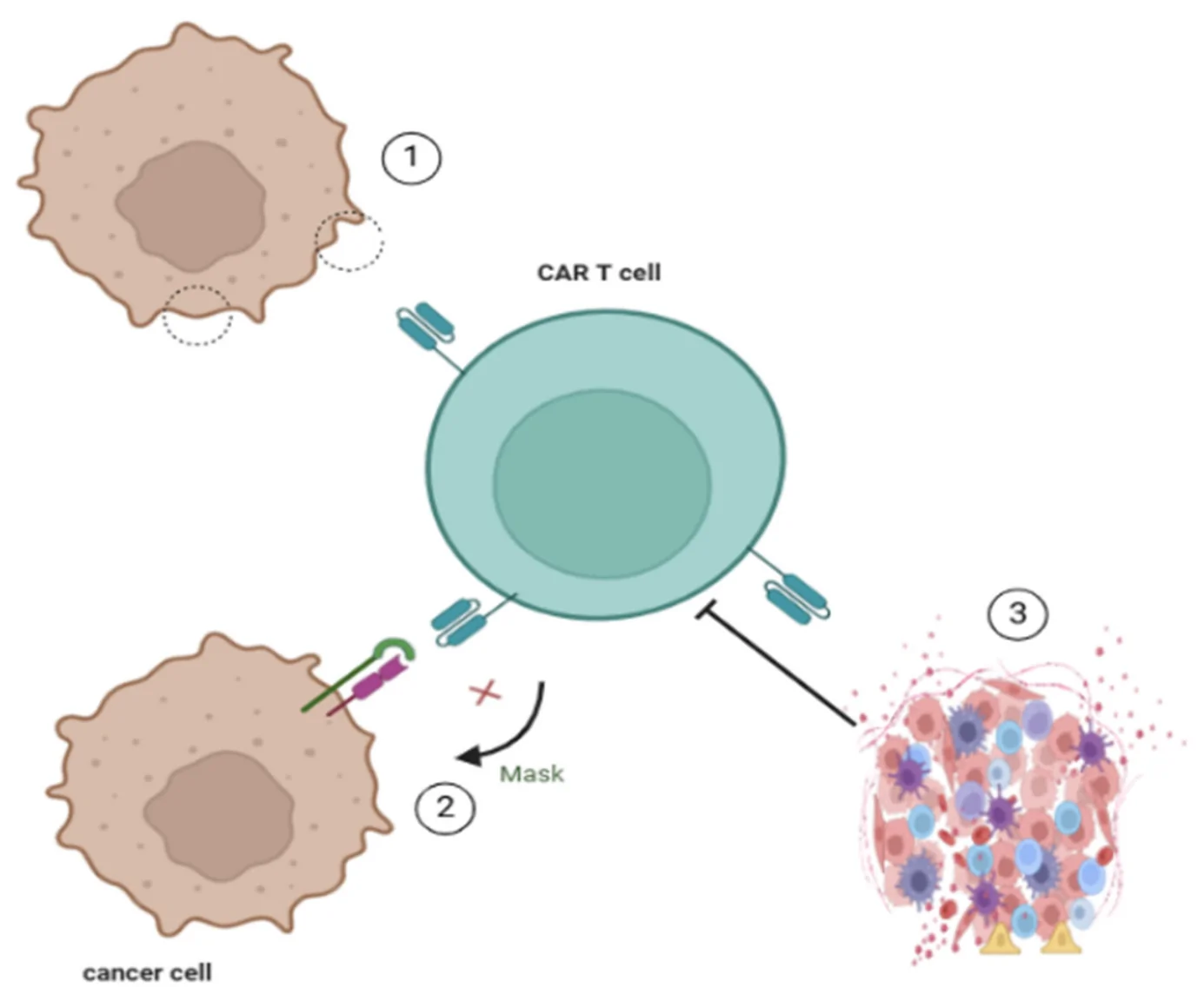
Tumor resistance mechanisms to CAR T-cells: (1) loss of surface antigen expression, (2) masking of target antigen, and (3) tumor immunosuppressive microenvironment. (Figure created with BioRender.com).

TIL therapy, which involves isolating and expanding tumor-reactive T-cells ex vivo before reinfusion, is often combined with immune checkpoint inhibitors to overcome T-cell exhaustion and suppressive signals within the tumor microenvironment [12,13,14,15]. Both CAR T-cell and TIL therapy require individualized ex vivo cell manufacturing and share resistance mechanisms, including antigen loss and tumor microenvironment-mediated immunosuppression (Figure 2), that parallel challenges discussed for TCEs later in this review (Section 8.1). TCEs offer a mechanistically related but logistically distinct alternative, redirecting a patient’s endogenous T-cell repertoire directly without requiring individualized cell engineering or ex vivo expansion.

The recent success of tarlatamab, a DLL3-targeted bispecific TCE in metastatic SCLC, exemplifies this potential with phase 2 trials reporting an ORR of approximately 40% and median PFS of 4.9 months in relapsed or refractory metastatic cases [16,17].

This approach addresses some limitations of cellular therapies by enhancing T-cell tumor infiltration in metastatic sites, particularly in SCLC, but its efficacy in metastatic NSCLC requires further validation through ongoing clinical trials. In this review, we focus on TCEs in metastatic lung cancer, providing an updated and lung cancer-specific synthesis that incorporates the phase 3 DeLLphi-304 OS data, the full FDA approval of tarlatamab, emerging CNS efficacy data, first-line maintenance results, the obrixtamig clinical program, and an expanded target pipeline—extending the scope of prior reviews of TCEs in solid cancers. We will explore the current progress, molecular and cellular mechanisms driving TCE efficacy, and future directions for optimizing these therapies to overcome resistance and improve outcomes in metastatic NSCLC and SCLC.

Despite this progress, several fundamental questions continue to shape the direction of TCE development in lung cancer and are addressed throughout this review: how target selection and antigen heterogeneity across SCLC and NSCLC subtypes should inform platform design; how engineering strategies, including half-life extension, CD3-affinity tuning, valency, and conditional activation, can mitigate on-target/off-tumor toxicity and cytokine release syndrome without compromising efficacy; what mechanisms underlie primary and acquired resistance to TCE therapy, and whether these are target-specific or shared across platforms; and how TCEs might be rationally combined with existing immunotherapy and chemotherapy backbones. Rather than cataloguing candidate targets and clinical programs in isolation, this review uses these questions as an organizing framework, with the Discussion and Conclusion returning to each explicitly. As will be shown, the available evidence speaks more directly to the first two questions, target selection and engineering strategy, than to the latter two, where resistance mechanisms remain incompletely characterized and combination data are still in early stages. This asymmetry is itself a key finding, highlighting where the field’s evidence base is most mature and where critical gaps remain.

## 2. Materials and Methods

We searched PubMed, Embase, and ClinicalTrials.gov from database inception through June 2026, supplemented by abstracts from the American Society of Clinical Oncology (ASCO), European Society for Medical Oncology (ESMO), American Association for Cancer Research (AACR), and American Thoracic Society (ATS) annual meetings.

Search terms were used individually and in combination: “T-cell engager”, “bispecific antibody”, “BiTE”, “thoracic malignancy”, “lung cancer”, “small cell lung cancer”, “SCLC”, “non-small cell lung cancer”, “NSCLC”, “tarlatamab”, “DLL3”, “DLL3/CD3”, “Claudin-18.2”, “TROP-2”, “FOLR1”, “CD70”, “CEACAM5”, “HER2”, “GPC3”, “VISTA”, “TIM-3”, “MUC1”, “CLDN6”, “B7-H4”, “PSMA”, “cytokine release syndrome”, “ICANS”, and “tumor microenvironment”.

We included peer-reviewed clinical and preclinical studies, phase 1–3 trial reports, FDA regulatory documents, systematic reviews, and conference abstracts from major oncology meetings. Non-English publications, single case reports, and editorials without original data were excluded unless they contained mechanistic or clinical information not otherwise available. All clinical trials referenced were independently verified on ClinicalTrials.gov for enrollment status and available results. Given the narrative review design, formal meta-analytic pooling was not performed; results are reported descriptively with original statistics preserved. Consistent with this narrative review design, formal PRISMA-compliant search methodology and a structured risk-of-bias assessment were not applied; this methodological limitation, and its implications for the certainty of conclusions drawn from the included evidence, is discussed further in Section 8.2.

Table 1 below summarizes the search strategy in a single, transparent reference. A formal PRISMA flow diagram with specific record counts at each screening stage is not provided, since this narrative review did not track those counts, consistent with the non-PRISMA methodology disclosed above and in Section 8.2.

## 3. Structural Design and Mechanism of Action of T-Cell Engagers

**Figure 3:**
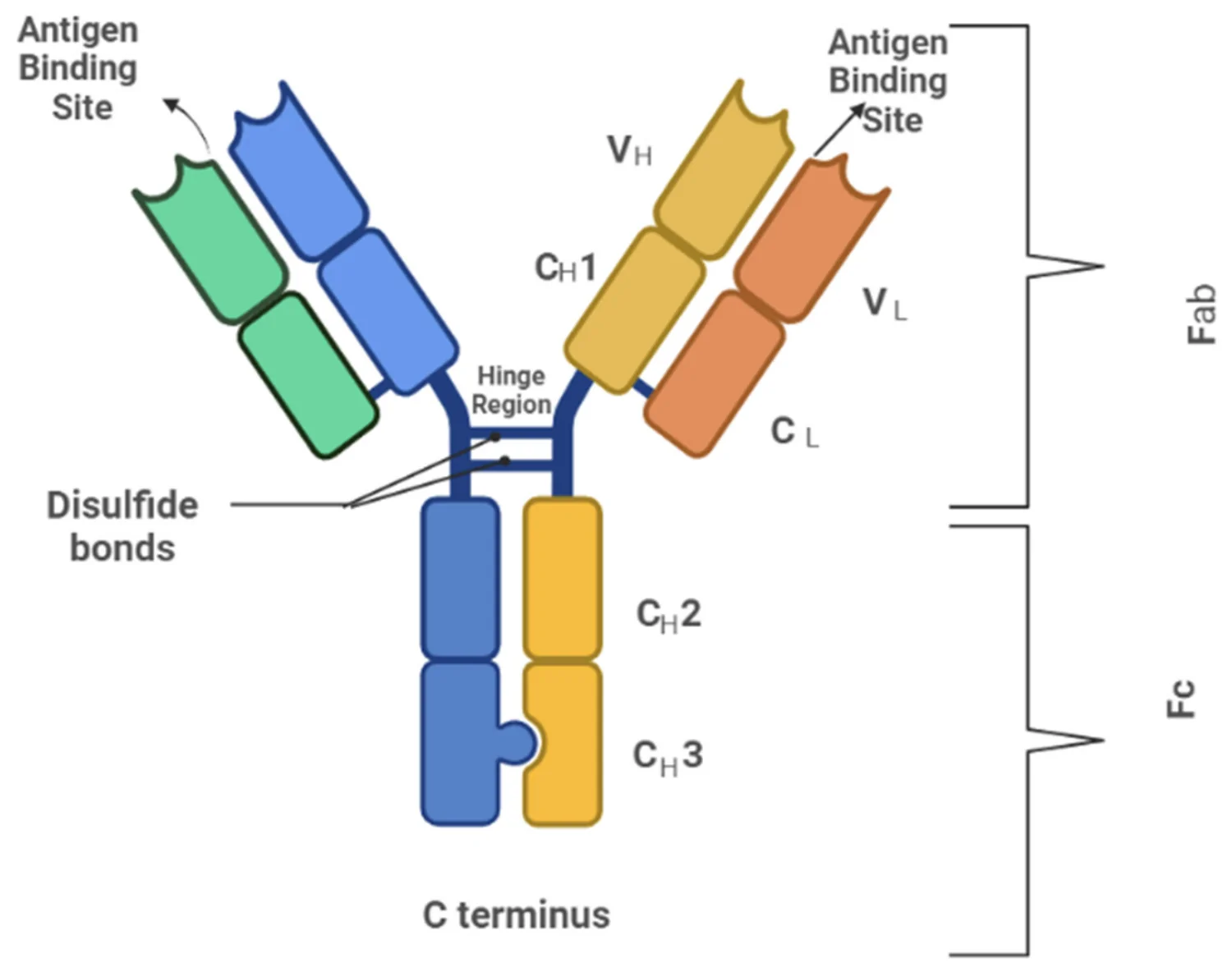
Basic BsAb structure showing the antigen-binding site harboring the complementarity-determining region (CDR) segment, disulfide bond, light chains (L) and heavy chains (H); C: constant domain; V: variable domain; Fab: fragment antigen-binding domain; Fc: fragment-crystallizable domain. (Figure created by BioRender.com).

Bispecific antibodies (BsAbs), as shown in Figure 3, are engineered by combining the specific binding domains of two distinct monoclonal antibodies into a single molecular construct, enabling simultaneous engagement of two different antigens and supporting diverse therapeutic strategies [18,19]. In the context of TCEs, this dual-targeting capability is exploited to bridge tumor cells and T-cells by binding a tumor-associated antigen (TAA) on the cancer cell surface and the CD3 epsilon subunit of the T-cell receptor complex, thereby inducing T-cell-mediated cytotoxicity independent of classical MHC-I antigen presentation.

Upon TCE binding, a cytolytic immunological synapse forms between the redirected T-cell and the tumor cell. This synapse promotes T-cell receptor (TCR) crosslinking and activation, triggering the release of perforins and granzymes that induce apoptosis in the targeted cancer cells, the secretion of pro-inflammatory cytokines driving T-cell proliferation, and downstream recruitment of additional effector immune cells. This mechanism is particularly relevant in solid tumor settings where MHC-I downregulation—a known immune escape mechanism—renders tumor cells invisible to conventional cytotoxic T-lymphocyte responses [14,15].

The majority of TCEs are derived from immunoglobulin G (IgG) molecules, with antigen-binding sites formed by the interaction of heavy variable (VH) and light variable (VL) chains shaped through the rearrangement of complementarity-determining regions (CDRs). BsAbs, including TCEs, are classified into two major structural categories based on the presence or absence of an Fc fragment, each with distinct pharmacokinetic and functional profiles (Figure 3).

### 3.1. T-Cell Engagers Without an Fc Fragment

**Figure 4:**
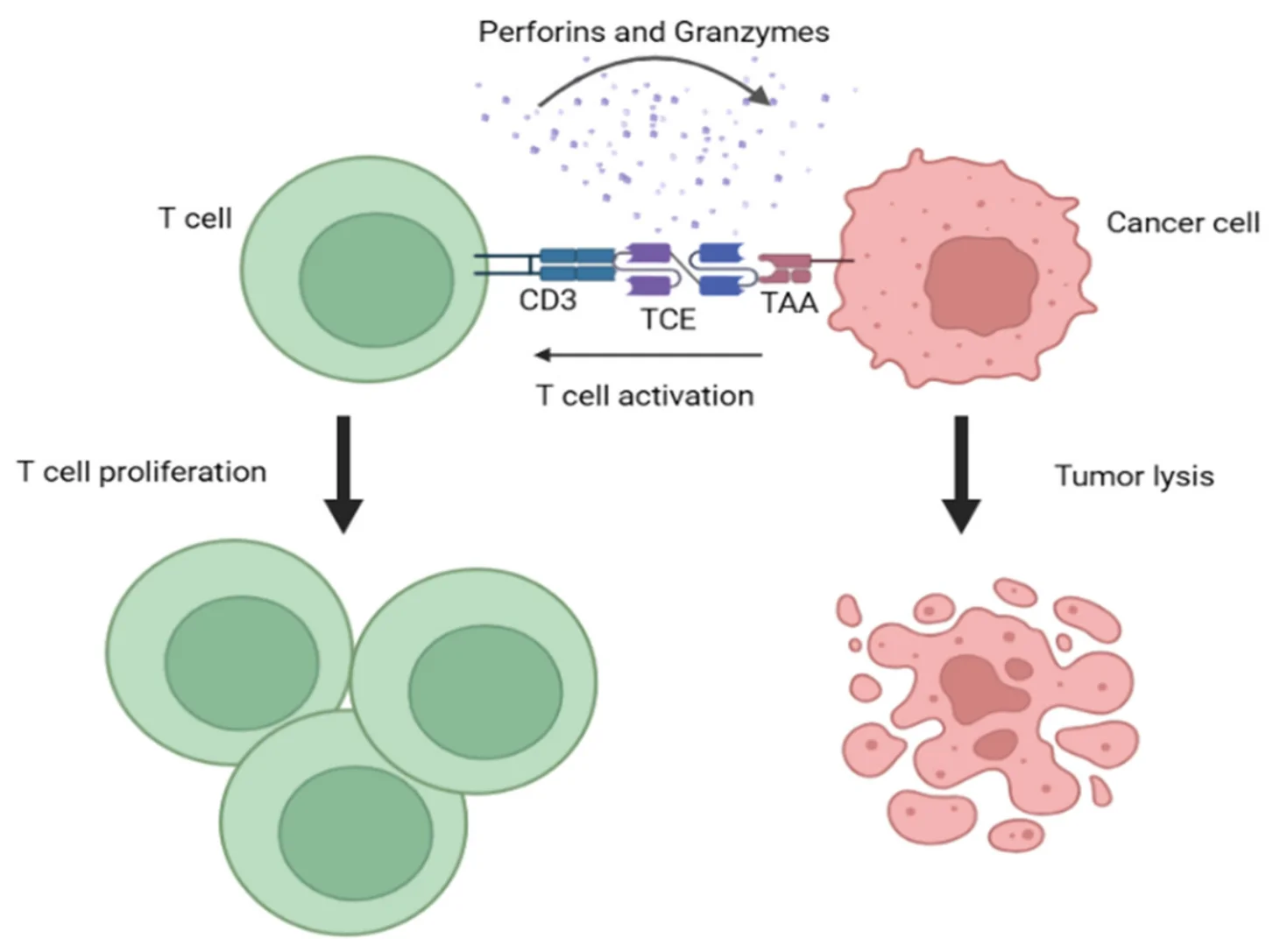
BsAbs without an Fc portion connected via a linker, demonstrating T-cell activation and tumor lysis through perforin and granzyme release. TAA, tumor-associated antigen. (Figure created with BioRender.com).

Fc-less TCEs depicted in Figure 4, such as blinatumomab, are compact bispecific constructs typically 50–60 kDa in molecular weight. Their smaller size confers superior tissue penetration and enhanced access to tumor cells in the solid tumor TME. However, the absence of an Fc domain results in rapid clearance, necessitating continuous intravenous infusion or highly frequent dosing schedules to maintain therapeutic concentrations. This rapid clearance occurs predominantly through renal filtration, since Fc-less constructs fall below the kidney’s molecular weight cutoff; lack of FcRn-mediated recycling, which normally salvages IgG-based molecules from degradation, is the major contributing factor, though FcRn-independent catabolic pathways also contribute and renal filtration would limit persistence even in a hypothetical construct with intact FcRn engagement. The absence of the Fc domain also precludes engagement of macrophages or natural killer (NK) cells via antibody-dependent cellular cytotoxicity (ADCC), making these constructs entirely reliant on T-cell activation for their antitumor effect. Ongoing research is exploring novel Fc-less formats—including nanobodies and DARTs—as well as advanced delivery systems such as nanoparticle encapsulation to improve stability and half-life in lung cancer.

### 3.2. T-Cell Engagers with an Fc Fragment

**Figure 5:**
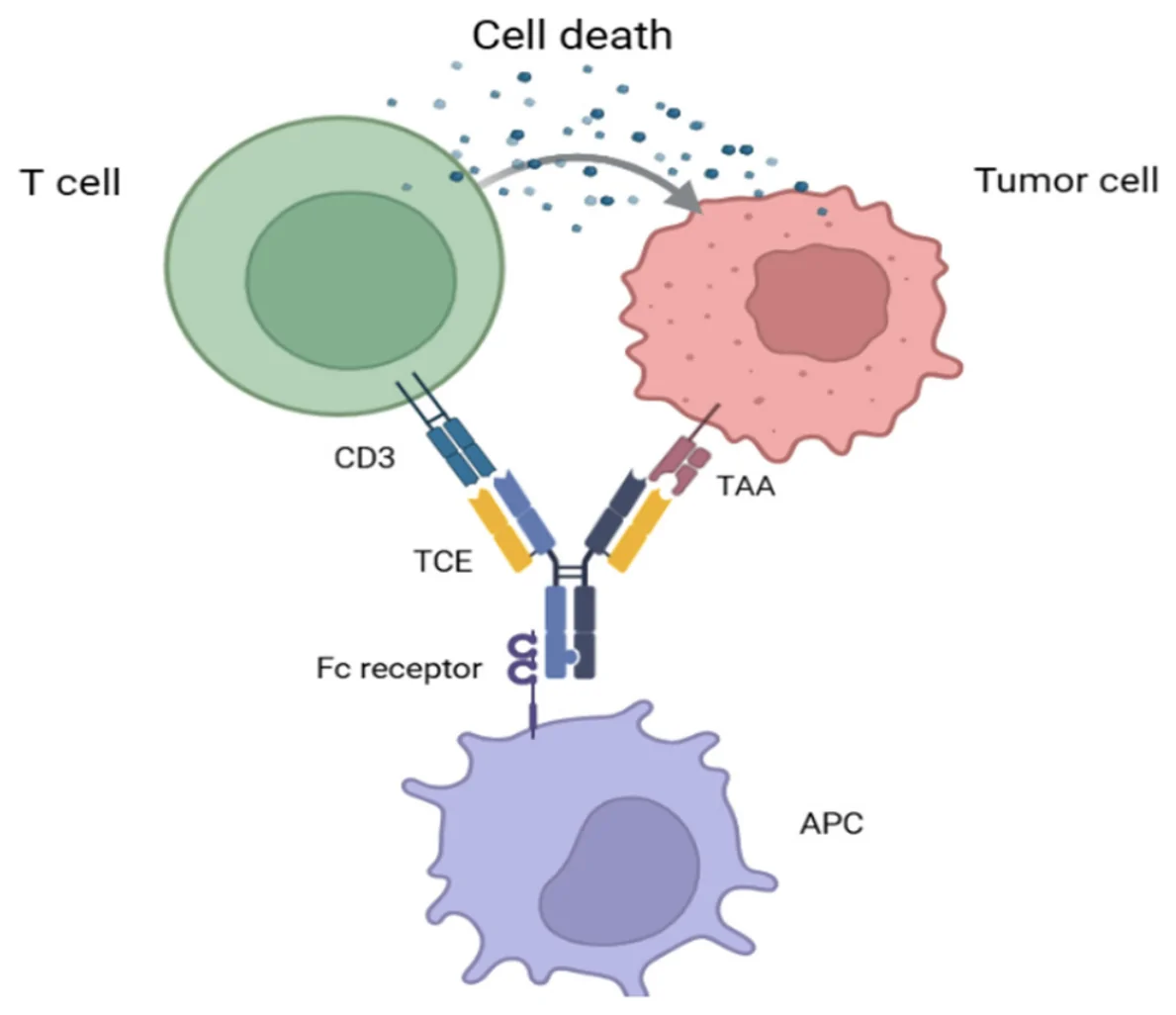
Classic BsAbs with an Fc portion that require antigen-presenting cells (APC) and Fc receptor engagement for full activation. CD3, cluster of differentiation 3; TAA, tumor-associated antigen; APC, antigen-presenting cell. (Figure created with BioRender.com).

TCEs incorporating an Fc fragment, as seen in Figure 5, benefit from extended plasma half-life and improved pharmacokinetic profiles through neonatal Fc receptor-mediated recycling, as well as the ability to engage Fc receptor-bearing immune cells including macrophages and NK cells via ADCC—a mechanism that can amplify antitumor activity. However, the larger molecular size can limit tissue penetration in metastatic settings. Additionally, intact Fc domains may impair T-cell trafficking and antitumor efficacy by engaging activating Fc receptors in an unintended manner. These limitations can be addressed through Fc silencing modifications—engineering strategies that abrogate effector function while preserving the pharmacokinetic benefits of the Fc domain. Tarlatamab exemplifies this approach: it incorporates an Fc domain to extend half-life while utilizing Fc silencing to minimize off-target Fc-mediated effects [15].

### 3.3. Advanced Engineering Strategies to Optimize Efficacy and Safety

The following engineering strategies directly address the second framework question posed in the Introduction: how design choices can mitigate toxicity and improve efficacy without one necessarily coming at the cost of the other. Beyond Fc engineering, several additional design strategies have emerged to address the core challenges of TCE therapy: target heterogeneity, on-target/off-tumor toxicity, limited tumor penetration, systemic T-cell activation, and T-cell exhaustion.

#### 3.3.1. CD-Affinity Optimization

Early TCE platforms used high-affinity CD3 binding domains to maximize T-cell engagement, but this approach is increasingly recognized as a double-edged strategy: high CD3 affinity promotes systemic T-cell activation independent of tumor antigen engagement, contributing to cytokine release syndrome even at low tumor burden [16,17,18,19,20]. Lowering CD3-arm affinity while preserving tumor-antigen-arm affinity shifts activation toward an avidity-driven mechanism, requiring simultaneous engagement of both tumor antigen and CD3 for productive signaling, thereby reducing off-tumor T-cell activation while preserving potency at the tumor site. This principle is exemplified by preclinical data showing that reducing CD3-arm affinity in FOLR1- and CEACAM5-targeting T-cell bispecifics maintained tumor cell killing while substantially reducing cytokine secretion, with the optimal CD3 affinity varying by target antigen [21].

#### 3.3.2. Valency and Geometry

Bivalent or trivalent tumor-antigen-binding arms increase avidity for antigen-low tumor cells, partially addressing the challenge of target heterogeneity within a single tumor [21,22]. However, increased valency can also increase steric bulk, reducing tumor penetration in the setting of solid, poorly vascularized metastatic deposits, and may theoretically increase the risk of antigen-independent T-cell clustering. Geometry, including the spatial orientation and interdomain spacing of the antigen-binding and CD3-binding domains, further influences immune synapse formation and cytotoxic efficiency independent of binding affinity alone; placing the tumor- and CD3-binding domains in a cis-configuration on the same arm has been shown to substantially enhance potency compared to a trans-configuration [23].

#### 3.3.3. Costimulatory Integration

Chronic antigen exposure in the tumor microenvironment can drive T-cell exhaustion, limiting the durability of TCE-mediated cytotoxicity [24]. Next-generation constructs are incorporating costimulatory domains, such as 4-1BB agonism, alongside the primary CD3-engaging arm to sustain T-cell proliferative capacity and delay exhaustion, an approach conceptually analogous to costimulatory signaling built into CAR-T constructs. This approach is exemplified by SAIL66, a CLDN6-targeting tri-specific TCE incorporating CD3/CD137 dual binding, discussed further in the Discussion (Section 8) [25], and by the EVOLVE platform, which integrates CD2 costimulation with attenuated CD3 binding to improve the therapeutic index [26].

#### 3.3.4. Conditionally Activated and Masked Platforms

To reduce systemic toxicity while preserving potency at the tumor site, several platforms now incorporate protease-cleavable masking domains that sterically block the CD3- or tumor-antigen-binding arm until cleaved by tumor-associated proteases enriched in the tumor microenvironment [27]. This conditional activation strategy is designed to concentrate T-cell engagement within the tumor and reduce systemic cytokine release, directly addressing the on-target/off-tumor toxicity that remains a major barrier to broader TCE adoption. The COBRA platform exemplifies this approach, demonstrating potent conditionally activated T-cell engagement in preclinical solid tumor models [28].

Collectively, these engineering strategies represent complementary, rather than mutually exclusive, approaches to the central design tension in TCE development: maximizing tumor-directed cytotoxicity while minimizing systemic toxicity and antigen-escape-driven resistance.

## 4. T-Cell Engagers in Metastatic Lung Malignancies

### 4.1. Tarlatamab (Imdelltra^®^): From Accelerated to Full FDA Approval

**Figure 6:**
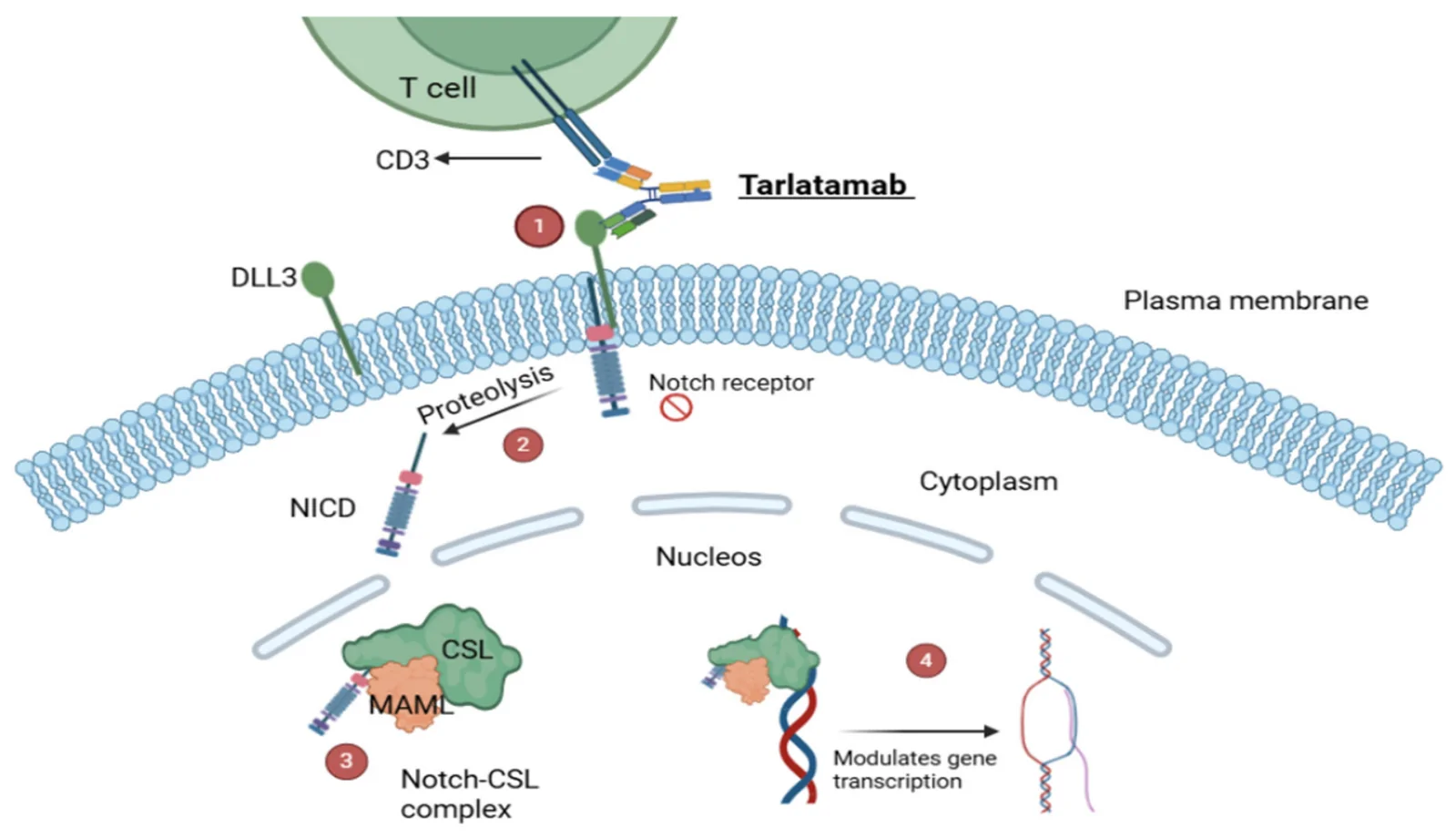
The Notch signaling pathway and its modulation by DLL3 binding and tarlatamab. DLL3 binding inhibits Notch signaling (1), leading to protease-mediated cleavage and release of the Notch intracellular domain (NICD) into the cytoplasm (2). NICD attaches to the transcription factor CBF1-suppressor of hairless-LAG1 (CSL) with the co-factor mastermind-like (MAML) (3), resulting in transcription of Notch pathway target genes (4). Tarlatamab simultaneously engages CD3 on T-cells and DLL3 on tumor cells to redirect cytotoxic T-cell killing. (Figure created by BioRender.com).

As of 2026, one TCE has received regulatory approval for metastatic lung malignancy, marking a significant milestone in systemic cancer therapy. Tarlatamab, a DLL3/CD3 bispecific TCE, was granted FDA approval in May 2024 for adults with ES-SCLC with disease progression following platinum-based chemotherapy. DLL3 is a Notch pathway inhibitory ligand, Figure 6, overexpressed in approximately 85–96% of SCLC cells, while minimally expressed in normal tissue, making it a highly tumor-selective target [15].

Tarlatamab’s regulatory journey began with accelerated FDA approval on 16 May 2024, based on the open-label phase 2 DeLLphi-301 (NCT05060016), which enrolled 220 patients with ES-SCLC across two dose cohorts (10 mg and 100 mg). The FDA accelerated approval was based on 99 efficacy-evaluable patients in the 10 mg cohort who had progressed following a median of two prior lines of systemic therapy. In this population, tarlatamab demonstrated an ORR of 40% (95% CI 31–51%), a median duration of response (DOR) of 9.7 months, and a median PFS of 4.9 months. The 100 mg group demonstrated an ORR of 32% [16,29,30]. These results were compelling in a population with extremely limited therapeutic options and historically low response rates to available second-line agents.

The definitive validation of tarlatamab’s clinical utility was established by the landmark phase 3 DeLLphi-304 (NCT05740566), which enrolled 509 patients with ES-SCLC following progression on platinum-based chemotherapy and randomized them 1:1 to receive tarlatamab (n = 254) or investigator’s choice of standard-of-care chemotherapy including topotecan, lurbinectedin, or amrubicin (n = 255) [17,31]. The context of these comparators matters: lurbinectedin, one of the three permitted control regimens, had itself failed to demonstrate an OS benefit in the confirmatory phase 3 ATLANTIS trial, and according to a company press release (non-peer-reviewed), also failed to meet its primary OS endpoint in the subsequent LAGOON; peer-reviewed publication of LAGOON data was pending at the time of submission [32,33]. The trial met its primary endpoint with a statistically significant and clinically meaningful improvement in overall survival: median OS of 13.6 months with tarlatamab versus 8.3 months with chemotherapy (HR 0.60; 95% CI 0.47–0.77; *p* < 0.001), representing a 40% reduction in the risk of death. Tarlatamab also demonstrated superior PFS, with a median of 4.2 versus 3.7 months. Because the proportional-hazards assumption was violated for this endpoint, restricted mean survival time (RMST) is emphasized here as the primary measure of treatment effect: at 12 months, RMST was 5.3 months with tarlatamab versus 4.3 months with chemotherapy, favoring tarlatamab [17]. The hazard ratio is reported only as supportive information given this methodological limitation, since HR loses its standard interpretation as a constant relative risk when the proportional-hazards assumption does not hold (HR 0.71; 95% CI 0.59–0.86; *p* = 0.002), a confirmed ORR of 35% versus 20%, a median DOR of 6.9 versus 5.5 months, and fewer grade ≥ 3 TRAEs (27% vs. 62%). Treatment-related discontinuation occurred in 3% of tarlatamab patients versus 6% of chemotherapy patients (per the ASCO abstract, which reports TRAE-attributed discontinuation); the NEJM publication reports a higher all-cause discontinuation rate of 5% versus 12%, which includes discontinuations attributable to disease progression and other reasons beyond TRAEs. Both figures are reported here for transparency, as they reflect different denominator definitions rather than a data inconsistency. Patient-reported outcomes showed clinically meaningful improvements in cancer-related symptoms including dyspnea and cough compared to chemotherapy. A Bayesian network meta-analysis subsequently confirmed tarlatamab’s OS superiority over all available second-line comparators—including platinum rechallenge in platinum-sensitive patients—with HRs ranging from 0.50 to 0.62, further establishing its position as the preferred second-line agent [34].

Based on these results, the FDA granted full traditional approval to tarlatamab on 19 November 2025, converting its prior accelerated approval status. The National Comprehensive Cancer Network (NCCN) subsequently listed tarlatamab as the only Category 1 preferred subsequent systemic therapy for ES-SCLC patients with disease progression on or after platinum-based chemotherapy. Notably, this Category 1 designation is unique to tarlatamab; other agents including topotecan, lurbinectedin, irinotecan, and platinum rechallenge remain listed as preferred options but at Category 2A designation, reflecting lower strength of evidence [35].

The intracranial activity of tarlatamab deserves specific attention, given that 40–70% of patients with ES-SCLC develop brain metastases [17]. A post hoc analysis of DeLLphi-304 evaluated CNS outcomes in 98 tarlatamab and 99 chemotherapy patients with baseline brain metastases. Among those with evaluable CNS imaging, tarlatamab numerically favored CNS PFS (median 6.5 vs. 4.2 months; HR 0.40; 95% CI 0.24–0.66), CNS complete response rate (15% vs. 5%), and CNS duration of disease control (8.2 vs. 5.2 months). Overall survival in the brain metastases subgroup also numerically favored tarlatamab (median OS 13.9 vs. 6.8 months; HR 0.51; 95% CI 0.34–0.74). These findings position tarlatamab as an effective systemic option that may spare patients the neurocognitive toxicity associated with whole brain radiation therapy in the relapsed setting [36].

Looking beyond second-line therapy, the phase 1b DeLLphi-303 (NCT05361395) [37] study evaluated tarlatamab 10 mg intravenously every 2 weeks in combination with an anti-PD-L1 antibody (atezolizumab 1680 mg or durvalumab 1500 mg every 4 weeks) as first-line maintenance therapy following platinum-etoposide chemo-immunotherapy in 88 patients with ES-SCLC. At a median follow-up of 18.4 months (IQR 15.2–23.0), median overall survival was 25.3 months (95% CI 20.3–not estimable) and median progression-free survival was 5.6 months. Because overall survival data were immature at the primary analysis, this estimate derives from a subsequent non-prespecified interim analysis, and the single-arm design precludes formal comparison with historical outcomes for PD-L1 inhibitor maintenance alone; the authors additionally note that exclusion of patients who progressed during first-line chemo-immunotherapy may have introduced attrition bias favouring survival. Serious adverse events occurred in 50 of 88 patients (57%), and cytokine release syndrome was the most common serious adverse event, reported in 21 of 88 patients (24%). Cytokine release syndrome events were predominantly low grade, most commonly manifesting as fever, occurred primarily after the 1 mg step dose or the first 10 mg dose, resolved in all affected patients, and did not lead to treatment discontinuation. Immune effector cell-associated neurotoxicity syndrome was reported as a serious adverse event in 4 of 88 patients (5%). The most frequent grade 3–4 adverse events were hyponatraemia (10%), anaemia (8%), and neutropenia (7%). No dose-limiting toxicities were observed, and no deaths were attributed to treatment-related adverse events. These safety and activity data provide the rationale for the ongoing randomised phase 3 DeLLphi-305 trial (NCT06211036), which is evaluating tarlatamab plus durvalumab versus durvalumab alone as first-line maintenance therapy after platinum-etoposide chemo-immunotherapy in ES-SCLC [37].

Despite its efficacy, tarlatamab’s use is tempered by common adverse events including cytokine-release syndrome (CRS) (51% in the 10 mg group, 61% in the 100 mg group), decreased appetite (29% and 44%, respectively), and pyrexia (35% and 33%, respectively), necessitating careful toxicity management in metastatic patients. In the phase 3 DeLLphi-304, CRS was predominantly low grade: grade 1 (42%), grade 2 (13%), and grade 3 (1%), confirming a manageable safety profile with appropriate premedication and monitoring [17,31].

### 4.2. Obrixtamig (BI 764532): The Emerging DLL3 Competitor

Beyond tarlatamab, obrixtamig (BI 764532; Boehringer Ingelheim, Ingelheim am Rhein, Germany) represents the most clinically advanced alternative DLL3-targeting TCE. Unlike tarlatamab’s half-life-extended BiTE architecture, obrixtamig is a DLL3/CD3 IgG-like TCE with an Fc-containing structure, allowing less frequent dosing without continuous infusion. Phase 1 dose-escalation results (NCT04429087) evaluated patients with previously treated DLL3-positive SCLC, large cell neuroendocrine carcinoma of the lung (LCNEC-L), and extrapulmonary neuroendocrine carcinomas across multiple dosing regimens. Among 168 patients treated (72% with ≥2 prior lines of therapy, 51% with prior anti-PD-1/PD-L1 therapy), obrixtamig at recommended regimens (B2/B3) demonstrated an ORR of 21% in SCLC, 27% in extrapulmonary NEC, and 70% in LCNEC-L patients, with a median duration of response of 8.5 months across all doses, regimens, and tumor types. CRS was the most common treatment-related adverse event (57% any grade, 3% grade ≥ 3) but was largely manageable with supportive care and anti-IL-6 receptor therapy; most cases occurred early and were reversible. Seven dose-limiting toxicities occurred during MTD evaluation (one in regimen A, six in regimen B2), and the maximum tolerated dose was not reached [38].

Direct comparison of tarlatamab and obrixtamig is informative, though it must be interpreted cautiously given the absence of head-to-head trial data and substantial differences in trial population and design, consistent with the cross-trial comparison limitations discussed in Section 8.2. Both agents target DLL3 and employ half-life extension strategies, yet their reported toxicity profiles differ modestly: tarlatamab’s any-grade CRS rate in the pivotal DeLLphi-304 trial (51% at the 10 mg approved dose) is somewhat lower than obrixtamig’s 57% any-grade CRS in its phase 1 dose-escalation cohort, while grade ≥ 3 CRS was similarly low for both agents (1% and 3%, respectively). This modest difference could plausibly reflect differences in CD3-binding domain affinity and overall molecular architecture between the two constructs. It could equally reflect differences in step-up dosing strategy, or simply the smaller, less mature dataset available for obrixtamig relative to tarlatamab’s phase 3 experience, and the current evidence cannot distinguish between these explanations. Efficacy comparison is even more limited by design heterogeneity: obrixtamig’s reported 21% ORR in SCLC reflects a phase 1 dose-escalation population that included patients across multiple prior treatment lines and dosing regimens, a substantially different trial design than the phase 3, randomized, dose-optimized DeLLphi-304 population from which tarlatamab’s efficacy data derive, precluding any meaningful claim of comparative efficacy at this stage. What can be concluded is that two independently developed DLL3/CD3 TCEs with similar overall architecture have each demonstrated a broadly consistent toxicity signature dominated by low-grade, manageable CRS, which lends some reassurance that this toxicity profile reflects a class effect of DLL3/CD3 engagement rather than an idiosyncratic feature of either individual construct.

Preliminary combination data from the phase 1b DAREON-9 (NCT05990738) showed that among 25 patients who received obrixtamig plus topotecan for relapsed/refractory SCLC, 23 evaluable patients had an unconfirmed ORR of 70% (95% CI 47–87%), including 1 complete response (4%) and 15 partial responses (65%), and a disease control rate of 87% (95% CI 66–97%); among the 13 patients with ≥2 post-baseline tumor assessments, the confirmed ORR was 69% (95% CI 39–91%). All CRS cases were low grade (grade 1 in 44%, grade 2 in 4%). The most common grade 3–4 adverse events, predominantly attributable to topotecan, included neutropenia (60%), thrombocytopenia (52%), decreased lymphocyte count (32%), and anemia (24%), and no obrixtamig- or topotecan-related grade ≥ 2 neurologic events occurred. These early results, while requiring validation in larger cohorts and longer follow-up, suggest that TCE and chemotherapy combinations may improve on topotecan monotherapy activity [39]. The phase 2 DAREON-5 study (NCT05882058) continues to evaluate obrixtamig monotherapy in a broader DLL3-positive SCLC and neuroendocrine carcinoma population.

The parallel development of tarlatamab and obrixtamig in SCLC, alongside ongoing combination studies—including obrixtamig plus the PD-1 inhibitor ezabenlimab (NCT05879978) and tarlatamab plus the TROP-2-targeting ADC sacituzumab govitecan (NCT07328490)—reflects the field’s recognition that single-agent TCE activity, while meaningful, may be substantially enhanced by mechanistically complementary partners that address TME suppression or deliver direct tumor cytotoxicity through independent pathways.

### 4.3. TCE Proof-of-Concept Across Solid Tumors: Lessons for Thoracic Oncology

The clinical validation of TCEs in solid tumors extends beyond SCLC. Tebentafusp, a gp100/HLA-A*02:01-targeting ImmTAC molecule, received FDA approval in January 2022 for metastatic uveal melanoma—the first TCE approval in any solid tumor. In its phase 3 trial (NCT03070392), tebentafusp significantly improved OS versus investigator’s choice, with 3-year follow-up confirming median OS of 21.6 versus 16.9 months (HR 0.68; 95% CI 0.54–0.87) despite a low conventional ORR (~5%), demonstrating that TCE-mediated clinical benefit can extend beyond RECIST-defined tumor shrinkage through immune priming and TME remodeling [40]. Xaluritamig (AMG 509), a STEAP1×CD3 XmAb 2+1 TCE targeting metastatic castration-resistant prostate cancer, has demonstrated an ORR of approximately 41% in high-dose cohorts in a phase 1 study (NCT04221542), with a phase 3 trial now active [41]. These cross-tumor clinical validations reinforce the generalizability of the TCE platform to solid tumors and provide molecular and mechanistic precedent for ongoing efforts to translate TCE efficacy into NSCLC, where the target landscape is broader, but the TME presents analogous challenges.

## 5. Potential Targets for Future TCE Development in Thoracic Malignancies

Beyond DLL3, a broad portfolio of tumor-associated antigens expressed in metastatic NSCLC and SCLC is under active investigation as TCE targets. We stratify these targets by level of evidence: clinical-stage targets with early-phase human trial data, and preclinical-stage targets currently supported only by in vitro or animal model data. For preclinical targets, activity signals are reported as tumor growth inhibition or cytotoxic T-cell killing rates from model systems—not as objective response rates (ORR), which is a clinical endpoint applicable only to human trials.

A third, intermediate category is also used: clinically validated targets with preclinical-stage TCE development. This category applies to antigens where tumor-selective expression has been independently validated through an approved or late-stage non-TCE modality, most often an antibody-drug conjugate, but where development of a TCE directed against that same antigen has not yet progressed beyond preclinical models. This distinction matters because clinical validation of a target’s expression pattern and safety profile in one therapeutic modality does not equate to clinical-stage development of a TCE against that target, which requires independent evidence given the mechanistic differences between modalities discussed above. Targets are assigned to this intermediate category, rather than to Section 5.1, specifically where no human trial data exist for a TCE directed at that antigen, regardless of how mature the non-TCE clinical evidence for the same target may be.

Throughout this section, several targets are described as supported in part by clinical data from approved or late-stage antibody-drug conjugates (ADCs) directed at the same antigen. This evidence should be interpreted as target-level, not modality-level, validation. ADCs and TCEs impose fundamentally different requirements on their shared antigen: ADCs depend on efficient receptor-mediated internalization to deliver a cytotoxic payload intracellularly, whereas TCEs require the antigen to remain stably expressed at the cell surface long enough to form a durable immune synapse, meaning that an antigen’s favorable internalization kinetics for ADC activity may be neutral, or even unfavorable, for TCE-mediated killing. The two modalities also tolerate different thresholds of normal-tissue expression, since ADC payload delivery is typically restricted to cells with sufficiently high receptor density for efficient uptake, while TCEs can activate cytotoxic T-cells against even modest levels of surface antigen on normal tissue, increasing the relative risk of on-target/off-tumor toxicity. Spatial accessibility of the antigen epitope, which matters less for ADC internalization than for the formation of a productive T-cell synapse, is a further independent variable. For these reasons, ADC clinical activity at a given antigen is discussed below as supportive context for tumor-selective expression, not as direct evidence of TCE efficacy or safety, both of which require independent validation specific to the TCE modality.

### 5.1. Clinical-Stage Targets

#### 5.1.1. Claudin-18.2 (CLDN18.2)

Claudin-18.2 is highly expressed on metastatic NSCLC cells but minimally present in normal lung tissue, making it a selective TCE target. Phase I clinical trials targeting CLDN18.2 in metastatic gastrointestinal cancers have reported ORRs of 15–25% in human patients, with preliminary activity signals in NSCLC. DR30318, a novel tri-specific TCE targeting CLDN18.2, has shown tumor growth inhibition in preclinical models [42]. CLDN18.2 expression correlates with increased T-cell infiltration in solid tumors, including pancreatic cancer, with preclinical evidence supporting similar immunomodulatory effects in NSCLC, suggesting it may enhance antitumor immunity [43]. Off-target toxicity and TME immunosuppression are ongoing challenges.

#### 5.1.2. Carcinoembryonic Antigen-Related Cell Adhesion Molecule 5 (CEACAM5)

CEACAM5 is overexpressed in metastatic NSCLC, with higher expression in non-squamous tumors harboring KRAS mutations and low PD-L1 expression [44,45]. Phase 1 trials of cibisatamab, a CEA/CEACAM5-CD3 bispecific TCE, have reported ORRs of 4% as monotherapy and 7% in combination with the PD-L1 inhibitor atezolizumab across CEA-positive solid tumors, predominantly colorectal cancer, with ORR up to 14% in a microsatellite-stable colorectal cancer subgroup receiving the combination [46]; NSCLC-specific efficacy data for a CEACAM5-targeting TCE have not yet been published. These figures should not be conflated with the CEACAM5-targeting antibody-drug conjugate tusamitamab ravtansine, which has shown substantially higher response rates in CEACAM5-high NSCLC but represents a distinct therapeutic modality. TME suppression and antigen shedding remain challenges for the TCE approach specifically. A landscape study in resected NSCLC confirms CEACAM5 expression in approximately 25% of lung adenocarcinoma cases, supporting target prevalence [45].

### 5.2. Clinically Validated Targets with Preclinical-Stage TCE Development

#### 5.2.1. Trophoblast Cell-Surface Antigen 2 (TROP-2)

TROP-2 is overexpressed in metastatic NSCLC and SCLC, where it drives tumor proliferation and is associated with poor prognosis. TROP-2′s tumor-selective expression has been independently demonstrated through strong ADC clinical activity, providing target-level, though not TCE-specific, supporting evidence: sacituzumab govitecan achieved an ORR of 17% and median OS of 9.5 months in metastatic NSCLC, and datopotamab deruxtecan (Dato-DXd) has shown activity across multiple clinical trials [46,47,48]. TCE preclinical models demonstrate T-cell-mediated cytotoxicity against TROP-2-expressing lung cancer cells with potential synergy with checkpoint inhibitors. Antigen shedding into the tumor microenvironment remains a key resistance mechanism [49].

#### 5.2.2. ErbB2 (HER2)

HER2 is overexpressed in a subset of metastatic NSCLC and is a validated target across multiple solid tumors. Clinical-stage TCE development at HER2 in thoracic malignancies remains early, with no published phase 1 results specific to lung cancer at the time of writing. The HER2-targeting ADC trastuzumab deruxtecan (T-DXd) has established robust HER2-directed antitumor activity in HER2-mutant and HER2-overexpressing NSCLC, confirming tumor-selective HER2 expression as a target-level rationale for TCE development at this antigen, though TCE-specific efficacy and toxicity remain to be independently established [50]. Antigen loss in metastatic lesions and cardiac toxicity risks require careful patient selection.

#### 5.2.3. Folate Receptor Alpha (FOLR1)

FOLR1 is overexpressed on a subset of NSCLC cells with limited normal tissue expression [51]. A phase 1/2 trial of PRO1184, a FOLR1-targeting ADC, is ongoing in metastatic solid tumors including NSCLC (NCT05547022), though preliminary results have not been published [51,52]. TCE development at FOLR1 in thoracic cancers remains preclinical; the favorable expression profile provides target-level rationale, and while ADC activity at this antigen offers additional supportive context, TCE-specific proof-of-concept in thoracic malignancy has not yet been established.

### 5.3. Preclinical-Stage Targets

The following targets are supported by in vitro and animal model data only; no clinical trial results in thoracic malignancies have been published. Activity signals are reported as T-cell cytotoxicity rates or tumor growth inhibition from preclinical systems and should not be interpreted as clinical efficacy data.

#### 5.3.1. CD70

CD70 is upregulated in metastatic SCLC and NSCLC, where it contributes to immune evasion, with minimal expression in normal lung tissue. CD70-targeting TCEs in preclinical lung cancer models demonstrate tumor-specific T-cell cytotoxicity, though MHC-I downregulation in metastatic lesions limits efficacy in animal systems [53,54]. No clinical trial results in thoracic malignancies have been reported.

#### 5.3.2. Glypican-3 (GPC3)

GPC3 is overexpressed in certain NSCLC and SCLC subtypes with minimal normal lung expression [55]. GPC3-targeting TCEs demonstrate antitumor T-cell cytotoxicity in preclinical lung cancer models, with ongoing efforts to overcome TME-induced resistance through combination with checkpoint inhibitors. No clinical trial data in thoracic malignancies are available.

#### 5.3.3. V-Domain Ig Suppressor of T-Cell Activation (VISTA)

VISTA is an immune checkpoint overexpressed on tumor cells and T-cells in metastatic SCLC, where it suppresses T-cell activity. VISTA/CD3 TCEs demonstrate tumor-specific T-cell cytotoxicity in preclinical SCLC models, with potential synergy with PD-1/PD-L1 inhibitors given overlapping checkpoint biology. No clinical trial data in thoracic malignancies exist to date.

#### 5.3.4. T-Cell Immunoglobulin and Mucin Domain-3 (TIM-3)

TIM-3 is highly expressed on exhausted T-cells and tumor cells in metastatic NSCLC and SCLC. TIM-3-targeting TCEs aim to restore T-cell function in the TME; preclinical models demonstrate T-cell reactivation with partial tumor growth inhibition. Combination with PD-1 or LAG-3 inhibitors is biologically rational given TIM-3′s co-expression with other exhaustion markers. No clinical data in thoracic malignancies have been published.

#### 5.3.5. MUC1

MUC1 is aberrantly glycosylated in lung cancer, exposing tumor-specific epitopes that distinguish it from normal tissue expression. MUC1 plays roles in tumor progression, metastasis, and immune evasion [56]. A MUC1-C/CD3 biparatopic bispecific TCE demonstrated effective lymphocyte cytotoxic function in MUC1-expressing breast cancer models, providing mechanistic rationale for extension to lung cancer [57].

#### 5.3.6. Claudin-6 (CLDN6)

CLDN6 is an oncofetal protein re-expressed in lung cancer but absent in healthy adult tissues, making it an ideal tumor-associated antigen [58]. SAIL66, a tri-specific TCE targeting CLDN6 via CD3 and CD137, demonstrated superior intratumoral T-cell infiltration, reduced T-cell exhaustion, and greater antitumor efficacy compared to conventional CLDN6 TCEs in tumor-bearing animal models [25].

#### 5.3.7. B7-H4

B7-H4 is overexpressed in NSCLC and regulates cancer stemness and epithelial-to-mesenchymal transition (EMT). Silencing B7-H4 in NSCLC cell lines inhibited EMT markers, enhanced E-cadherin expression, and reduced cell migration through the AMPK/mTOR pathway, positioning it as a promising TCE target in NSCLC [59].

#### 5.3.8. Prostate-Specific Membrane Antigen (PSMA)

PSMA is expressed in tumor neovasculature endothelial cells across solid tumors and in the cancer cells of approximately 54% of NSCLC patients, with higher rates in patients under 60 years [60,61]. CC-1, a PSMAxCD3 TCE, is under clinical evaluation in squamous cell lung cancer (NCT04496674), representing a transition toward early clinical investigation for this target.

### 5.4. DLL3 Testing: Practical Considerations for Patient Selection

Given the biomarker data from the Cancer Discovery 2026 study demonstrating that pretreatment DLL3 expression on CTCs predicts tarlatamab benefit with 85% sensitivity and 100% specificity, the question of routine DLL3 testing has direct clinical relevance [62]. DLL3 protein expression can be assessed by immunohistochemistry (IHC) on tumor tissue, and near-universal expression has been documented in ES-SCLC biopsies—a finding that formed the basis for tarlatamab’s approval without a mandatory DLL3 testing requirement. The current FDA label and NCCN guidelines do not require DLL3 IHC testing prior to tarlatamab initiation, reflecting the high background prevalence of expression in SCLC. However, the CTC-based DLL3 assay offers a non-invasive, dynamic, and quantitative alternative that captures both baseline expression and longitudinal changes—particularly the emergence of DLL3-low phenotypes that characterize acquired resistance. As liquid biopsy platforms continue to mature, prospective validation of a CTC-DLL3 threshold for patient selection in SCLC and extension of DLL3 testing to NSCLC—where expression is more heterogeneous—will be important priorities for clinical trial design and eventual regulatory consideration.

## 6. T-Cell Engagers Currently Under Clinical Evaluation in Thoracic Tumors

T-cell engagers under clinical evaluation in thoracic tumors were identified through a comprehensive search of PubMed, major oncology meeting abstracts, and ClinicalTrials.gov. Table 2 summarizes agents with available or pending clinical data.

Table 3 below provides a direct comparative overview of the three most clinically advanced DLL3/CD3 TCE platforms discussed above, consolidating their format, pharmacokinetic mechanism, dosing, toxicity, and clinical maturity into a single reference.

This comparison highlights that half-life extension can be achieved through mechanistically distinct strategies, FcRn-mediated recycling for tarlatamab and obrixtamig, versus an albumin-binding domain for HPN328, despite all three sharing the same DLL3/CD3 targeting logic. It also makes explicit where the evidence base remains immature, particularly for HPN328, where detailed toxicity and dosing data are not yet available in peer-reviewed form, underscoring that clinical maturity varies substantially even among agents targeting the same antigen.

## 7. T-Cell Engager-Associated Adverse Events and Toxicity Management

TCEs are associated with a distinct and class-specific toxicity profile driven by their mechanism of systemic T-cell activation and cytokine release. The most clinically significant adverse events include CRS, ICANS, infections, tumor flare reactions, and cytopenias [63].

### 7.1. Cytokine Release Syndrome (CRS)

CRS results from the rapid, systemic release of pro-inflammatory cytokines following TCE-mediated T-cell activation. Clinical manifestations range from grade 1–2 symptoms including fever, fatigue, and myalgias, to severe life-threatening grade 3–4 complications including hypotension, respiratory distress, acute heart failure, disseminated intravascular coagulation, and multi-organ dysfunction [64]. CRS typically occurs within the first 24–48 h of drug administration and is graded using the ASTCT consensus grading system [64,65].

Per the FDA-approved tarlatamab label, a mandatory step-up dosing protocol is required: patients receive an initial dose of 1 mg IV on day 1 of cycle 1, followed by the full therapeutic dose of 10 mg IV on day 8, and then 10 mg every two weeks thereafter. This step-up design substantially reduces the risk of severe CRS by allowing initial low-level T-cell activation before the full therapeutic exposure. Recommended premedication includes an antihistamine and antipyretic administered 30–60 min prior to each infusion during cycle 1, with corticosteroid premedication reserved for patients who experienced grade ≥ 2 CRS on a prior dose. Per the label, patients must be monitored for a minimum of 3 h following the 1 mg step-up dose and the first 10 mg dose, and for at least 30 min following subsequent doses, in a setting with immediate access to resuscitative measures. Treatment of established CRS employs corticosteroids or IL-6 pathway inhibitors—particularly tocilizumab—which have demonstrated rapid resolution of CRS in both clinical trial and real-world settings [63,64,65].

### 7.2. Immune Effector Cell-Associated Neurotoxicity Syndrome (ICANS)

ICANS frequently follows or co-occurs with CRS, with accumulating evidence suggesting that CRS-driven endothelial activation and blood–brain barrier disruption by secreted pro-inflammatory cytokines contribute to direct neuronal injury and the neurological manifestations of ICANS [24]. Clinical features range from mild presentations including confusion, headache, attention deficits, and focal neurological deficits to severe and life-threatening events including seizures, coma, and cerebral edema [24,65].

ICANS is primarily managed with supportive care for mild cases, while moderate-to-severe presentations require systemic corticosteroids—preferably dexamethasone given its favorable CNS penetration and ability to cross the blood–brain barrier. Close neurological monitoring during and after TCE administration is essential, and all patients should receive structured neurocognitive assessment using validated tools such as the CARTOX-10 or ICE scoring systems [24,65,66].

The grade-based management framework below consolidates the CRS and ICANS management guidance described above into a single structured reference, shown in Table 4.

## 8. Discussion

The emergence of T-cell engagers as a validated therapeutic platform in lung cancer represents one of the most significant immunotherapeutic advances since the introduction of immune checkpoint inhibitors. Tarlatamab’s full FDA approval [30] and NCCN Category 1 designation [35] establish a new standard of care in ES-SCLC—a disease where meaningful progress had been largely absent for three decades [17].

This review extends beyond prior TCE reviews in solid cancers by focusing specifically on thoracic malignancies and incorporating the landmark DeLLphi-304 phase 3 OS data, the full FDA approval of tarlatamab, CNS efficacy data, first-line maintenance trial results, the emerging obrixtamig clinical program, and an expanded target pipeline of 14 antigens with mechanistic rationale for lung cancer—content that was either unavailable or not covered in scope in earlier reviews. As discussed in Section 4.1, the DeLLphi-304 phase 3 data demonstrate not only survival superiority over chemotherapy but a more favorable toxicity profile and clinically meaningful patient-reported outcome improvements, collectively validating the TCE platform for solid tumors beyond the hematologic settings where bispecific antibodies first gained traction [16].

Several mechanistic features distinguish TCEs from prior immunotherapeutic strategies and explain their particular utility in the SCLC setting. First, the near-universal expression of DLL3 across ES-SCLC tumor cells (~85–96%) provides an exceptionally high target coverage rate rarely seen with other solid tumor antigens, minimizing the impact of antigen heterogeneity on response rates. Second, TCE-mediated T-cell cytotoxicity operates independently of MHC-I antigen presentation, a mechanism that SCLC tumors frequently downregulate as an immune escape strategy. Third, the Fc-silenced, IgG-based architecture of tarlatamab confers a favorable pharmacokinetic profile with manageable toxicity compared to Fc-less formats requiring continuous infusion [15].

It is important to distinguish the evidence tier of the discussion that follows from the Phase 3, FDA-approved data on tarlatamab described above: the broader pipeline of emerging TCE targets described below is supported predominantly by early-phase clinical or preclinical data and should be read as a description of a developing field rather than as established clinical practice. The broader pipeline of emerging TCE targets in thoracic malignancies—including CLDN18.2, TROP-2, HER2, CEACAM5, FOLR1, and CD70—reflects the field’s growing capacity to identify and exploit tumor-selective antigen expression profiles in NSCLC and SCLC. However, several shared resistance mechanisms present persistent challenges across this target landscape. TME-mediated immunosuppression, driven by regulatory T-cells (Tregs), myeloid-derived suppressor cells (MDSCs), and suppressive cytokines such as TGF-β and IL-10, continue to limit T-cell infiltration and persistence in solid tumors. Antigen shedding—where soluble forms of target antigens in the tumor microenvironment competitively bind and neutralize TCEs before they reach the tumor cell surface—remains a particular challenge for TROP-2, CEACAM5, and similar targets. Antigen heterogeneity, where only a subset of tumor cells express the target antigen at sufficient density, limits the depth and durability of response for most emerging targets, motivating the development of multi-specific TCE formats and combination strategies.

The development of tri-specific TCEs represents a particularly promising direction for overcoming these limitations. Agents such as DR30318 (targeting CLDN18.2) and SAIL66 (targeting CLDN6 with CD3/CD137 co-stimulation) exemplify how next-generation molecular engineering can simultaneously address tumor targeting, T-cell co-stimulation, and resistance to exhaustion within a single therapeutic construct [25,42]. SAIL66′s ability to reduce T-cell exhaustion while enhancing intratumoral infiltration—compared to conventional bispecific CLDN6 TCEs—illustrates the value of incorporating co-stimulatory signaling into TCE design.

Combination strategies pairing TCEs with immune checkpoint inhibitors represent another rational approach to overcoming TME-mediated resistance. The mechanistic complementarity is compelling: ICIs relieve checkpoint-mediated T-cell exhaustion and restore effector function, while TCEs redirect activated T-cells to the tumor cell surface. This rationale is now being tested clinically: obrixtamig combined with the PD-1 inhibitor ezabenlimab (NCT05879978) is in active phase 1 dose-escalation, and tarlatamab combined with the TROP-2-targeting ADC sacituzumab govitecan (NCT07328490) is enrolling in an NCI-sponsored phase 1/2 study for ES-SCLC and extrapulmonary neuroendocrine carcinoma. Notably, the combination of obrixtamig with topotecan (DAREON-9) produced a confirmed ORR of 69% in relapsed SCLC—substantially higher than either agent alone—providing early clinical evidence that TCE-chemotherapy combinations may be among the most promising strategies to meaningfully deepen and broaden response [39]. Preclinical models across multiple target-tumor combinations have demonstrated synergistic or additive antitumor activity, and early-phase clinical data from these trials are awaited with significant interest.

Similarly, combinations with epigenetic modulators—agents that upregulate antigen expression and enhance MHC-I surface density—represent an additional strategy to maximize TCE efficacy in antigen-low or antigen-heterogeneous tumors.

From a toxicity perspective, the management of CRS and ICANS has become increasingly refined as TCEs have moved through clinical development. The standardization of ASTCT consensus grading, the routine use of prophylactic premedication protocols, and the availability of effective rescue therapies including tocilizumab have substantially reduced the incidence of severe toxicity in recent trials. Tarlatamab’s phase 3 data demonstrating fewer grade ≥ 3 adverse events than chemotherapy represents an important clinical validation that TCE-associated toxicity, when well-managed, does not preclude favorable benefit-risk profiles in the metastatic setting.

The NSCLC TCE pipeline requires contextual framing relative to the existing treatment landscape. Unlike SCLC, metastatic NSCLC already has multiple approved and highly effective targeted therapies—EGFR/ALK/ROS1/KRAS TKIs in genomically defined subsets, and HER2-targeting ADCs including trastuzumab deruxtecan in HER2-altered tumors. TCEs in NSCLC are therefore most likely to find their role in patients whose tumors lack actionable genomic drivers and who have progressed on or are ineligible for existing regimens. In this biomarker-negative population, which represents approximately 40–50% of metastatic NSCLC, available second-line options remain limited to chemotherapy and immunotherapy combinations with modest response rates. TCEs targeting CLDN18.2, TROP-2, CEACAM5, and HER2 would compete and potentially synergize with their ADC counterparts, which have already demonstrated clinical validation at these same targets—and represent a mechanistically complementary approach that exploits T-cell cytotoxicity rather than drug payload delivery.

The key differentiating question for TCEs in NSCLC is not whether the target is expressed, but whether the TME is sufficiently immunologically active to support redirected T-cell killing—a question that will require careful patient stratification by tumor microenvironment phenotype in future trials.

A prospective cohort study published in Cancer Discovery 2026 demonstrated that pretreatment DLL3 expression on circulating tumor cells (CTCs) predicts tarlatamab clinical benefit with 85% sensitivity and 100% specificity in 20 patients with ES-SCLC [62]. Patients classified as DLL3-positive via CTC analysis had a 55% partial response rate and 45% stable disease rate, while DLL3-low patients rarely derived benefit. The same study characterized two distinct mechanisms of acquired resistance: loss of DLL3 expression with persistence of other targetable neuroendocrine epitopes, and persistence of DLL3 on CTCs accompanied by systemic markers of T-cell dysfunction [62].

These findings have direct implications for patient selection and combination strategy design: DLL3-low patients may be poor candidates for tarlatamab monotherapy, while those developing T-cell exhaustion-mediated resistance may benefit from the addition of checkpoint inhibitors or co-stimulatory agents. Integration of CTC-based biomarker assessment and longitudinal resistance monitoring into future clinical trial design will be essential for realizing the full potential of TCE therapy in SCLC.

Emerging real-world data are beginning to refine the clinical toxicity profile of tarlatamab beyond what controlled trial conditions capture. Blocker et al. (Eur J Cancer 2026) analyzed 109 patients at nine cancer centers and identified high disease burden (*p* = 0.002), hepatic disease (*p* = 0.002), and baseline oxygen use (*p* = 0.034) as statistically significant risk factors for any-grade CRS; high disease burden (*p* = 0.022) and pre-existing CNS disease were independently associated with ICANS [67]. A separate real-world series from Moffitt Cancer Center reported ICANS rates of 23% and CRS rates of 53% in patients not receiving prophylactic tocilizumab, with prophylactic tocilizumab use associated with CRS in only 10% and no ICANS events, supporting its consideration for high-risk patients. These data underscore the importance of structured toxicity monitoring and risk-stratified premedication protocols, particularly for patients with baseline CNS disease, high tumor burden, or hepatic involvement [66,67].

### 8.1. Common and Target-Specific Resistance Pathways

Resistance mechanisms to TCE therapy have been characterized in detail for tarlatamab and DLL3, primarily through circulating tumor cell (CTC)-based biomarker studies, but the broader literature also describes resistance-related phenomena for other targets discussed throughout this review, including antigen shedding for TROP-2 and CEACAM5, tumor microenvironment (TME) immunosuppression, antigen heterogeneity, and MHC-I downregulation. These mechanisms have not been systematically organized by whether they are platform-common or target-specific. Table 5 below consolidates this evidence.

The DLL3-specific data are the most mature: a prospective cohort study characterized two distinct resistance phenotypes in tarlatamab-treated patients, loss of DLL3 expression with lineage plasticity toward a YAP1-driven phenotype, and persistence of DLL3 accompanied by systemic T-cell exhaustion markers (PD-1, TIGIT, LAG-3 upregulation), as detailed in Section 5.4 and Section 6. The first of these, antigen loss with lineage plasticity, is inherently target-specific, since it depends on the particular biology of DLL3 and SCLC lineage plasticity and cannot be assumed to generalize directly to epithelial NSCLC targets such as TROP-2 or CEACAM5. The second, T-cell exhaustion, is mechanistically generic to any T-cell-dependent therapy and is reasonably expected across the TCE platform, though target-specific confirmatory data outside DLL3 are currently lacking.

Antigen shedding, by contrast, is a mechanism specific to targets whose extracellular domain can be proteolytically cleaved into a soluble form, currently documented for TROP-2 and CEACAM5 but not established for DLL3. TME-mediated immunosuppression and antigen heterogeneity are best characterized as common mechanisms affecting the TCE platform broadly, though their severity varies by target based on baseline expression uniformity and microenvironmental composition. MHC-I downregulation is included in Table 5 as an indirect, rather than direct, TCE resistance mechanism: because TCE-mediated cytotoxicity operates independently of MHC-I antigen presentation, MHC-I loss does not impair TCE activity the way it impairs T-cell receptor-based immunotherapies, and its inclusion here instead reflects its relevance to combination rationale in tumors that have already escaped MHC-I-dependent immune surveillance. Physical or stromal exclusion of T-cells, whereby dense desmoplastic stroma or aberrant tumor vasculature limits T-cell trafficking into the tumor core independent of antigen expression, represents an additional common mechanism relevant across the TCE platform and is included in Table 5 accordingly [68].

**Table 5:** Common and target-specific resistance pathways in TCE therapy for thoracic malignancies. Mechanisms are classified as “common” where they are expected to apply broadly across TCE targets based on shared immunologic principles, or “target-specific” where current evidence links them to particular antigens.

Resistance Mechanism	Category	Affected Targets	Clinical Implication
Antigen loss with lineage plasticity	Target-specific	DLL3 (documented); plausible for other lineage-dependent targets	Loss of surface antigen renders the TCE inactive regardless of T-cell fitness; supports serial biomarker monitoring rather than one-time testing
T-cell exhaustion (PD-1/TIGIT/LAG-3 upregulation)	Common	Documented for DLL3; mechanistically expected across all TCE targets	Antigen-independent resistance driven by chronic T-cell activation; rationalizes combination with checkpoint inhibitors or costimulatory TCE platforms
TME-mediated immunosuppression (Tregs, MDSCs, TGF-β, IL-10)	Common	All targets discussed in this review	Limits T-cell infiltration and persistence independent of antigen status; supports combination strategies targeting the microenvironment rather than the tumor cell alone
Antigen shedding	Target-specific	TROP-2, CEACAM5	Soluble antigen competitively neutralizes TCE before tumor engagement; may require shed-antigen-resistant construct design or antigen-level monitoring
Antigen heterogeneity (subclonal expression)	Common, with variable severity by target	Most emerging targets; least relevant for DLL3 given near-universal SCLC expression	Limits depth and durability of response; motivates multi-specific TCE formats and combination approaches
MHC-I downregulation	Common (indirect)	Broadly relevant across NSCLC and SCLC as an immune escape strategy	Does not directly impair TCE activity, since TCEs act independently of MHC-I; reinforces TCE mechanistic rationale in tumors already resistant to MHC-I-dependent immunotherapy
Physical/stromal exclusion	Common	Broadly relevant across desmoplastic and poorly vascularized tumors	Limits T-cell trafficking into the tumor core independent of antigen expression; supports strategies combining TCEs with stromal-modulating or anti-angiogenic agents
Antigen density (low receptor copy number)	Common	Broadly relevant; threshold varies by construct and target	Limits productive immune synapse formation below a minimum receptor copy number, independent of clonal heterogeneity; supports affinity/valency optimization or antigen-density-based patient selection [69]
Adaptive resistance (IFN-γ/JAK2 pathway disruption)	Common	Documented for HER2- and CEACAM5-directed TCEs; mechanistically plausible broadly	Reversible, therapy-induced disruption of interferon-gamma signaling reduces tumor susceptibility to T-cell killing without permanent antigen loss; supports combination strategies restoring IFN-γ responsiveness [70]

This framework has direct implications for combination strategy design: T-cell exhaustion and TME immunosuppression, as common mechanisms, rationalize broadly applicable combination approaches such as checkpoint inhibitor pairing or costimulatory TCE platforms (Section 3.3), whereas antigen loss and antigen shedding, as target-specific mechanisms, may instead require target-level solutions such as multi-specific constructs or biomarker-guided re-selection of therapy.

The mechanisms summarized in Table 5 can be understood collectively through the lens of cancer immunoediting, the process by which sustained immune pressure selects for tumor clones capable of evading detection, proceeding through elimination, equilibrium, and escape phases [71]. Acquired resistance mechanisms such as antigen loss with lineage plasticity and adaptive IFN-γ pathway disruption represent tumor-intrinsic escape strategies selected under TCE-mediated immune pressure, while TME-mediated immunosuppression and stromal exclusion represent complementary, tumor-extrinsic escape routes. Viewing resistance through this unifying framework, rather than as a list of unrelated phenomena, clarifies why combination strategies pairing TCEs with agents that restore immune visibility or T-cell function are mechanistically rational rather than empirical.

### 8.2. Limitations

Several limitations apply to this review and should be considered when interpreting its conclusions. First, as a narrative rather than systematic review, it is subject to selection bias in source identification and cannot claim exhaustive coverage of all published or conference-presented data; formal PRISMA-compliant search methodology and risk-of-bias assessment were not applied. Second, the evidence base for the majority of targets discussed beyond DLL3 remains limited to early-phase trials, in vitro models, or animal studies, which substantially constrain the certainty of any clinical efficacy conclusions drawn for these agents. Third, the literature search was conducted through June 2026; trial results, regulatory decisions, and guideline updates published after this date are not reflected. Fourth, one cited source is a non-peer-reviewed company press release; while appropriately flagged throughout, this claim should be interpreted with caution until peer-reviewed publication [33]. Fifth, direct cross-trial comparisons are constrained by heterogeneity in patient populations, prior lines of therapy, performance status criteria, response assessment methods, and follow-up duration, all of which affect reported outcomes independently of the therapeutic agents evaluated. Sixth, this review does not incorporate patient-reported outcome measures or health-economic analyses, which will be critical for future formulary and access decisions surrounding tarlatamab and next-generation TCEs.

## 9. Conclusions

T-cell engagers have established themselves as a clinically validated and scientifically compelling immunotherapeutic platform for lung cancer. The full FDA approval of tarlatamab for ES-SCLC, supported by phase 3 data demonstrating superior overall survival, improved progression-free survival, better patient-reported outcomes, and a more favorable safety profile than standard chemotherapy, marks a transformative milestone in the treatment of one of oncology’s most challenging diseases. The mechanistic advantages of TCEs, including MHC-I-independent cytotoxicity, tumor-selective antigen targeting, and flexible molecular engineering, position them as a durable component of the therapeutic armamentarium for lung cancer.

The emerging pipeline of TCE targets in metastatic NSCLC and SCLC, spanning CLDN18.2, TROP-2, FOLR1, CD70, CEACAM5, HER2, VISTA, TIM-3, MUC1, CLDN6, B7-H4, and PSMA, reflects both the breadth of antigenic opportunities in thoracic tumors and the substantial unmet need remaining for patients whose disease progresses beyond first-line therapy. Overcoming the shared resistance mechanisms outlined in Section 8.1 will require continued development of next-generation TCE formats, rationally designed combination regimens, and multi-omics-driven patient selection strategies. As the field progresses from proof-of-concept to practice-changing evidence, three priorities will determine how fully this potential is realized: validated predictive biomarkers, such as CTC-based DLL3 assessment, to guide patient selection beyond DLL3 in SCLC; real-world evidence generation to confirm that the efficacy and toxicity profiles observed in controlled trials translate to broader, more heterogeneous patient populations; and health-economic and cost-effectiveness analyses, which will be essential for formulary decisions and equitable access as next-generation TCEs and combination regimens enter clinical practice. The momentum established by tarlatamab’s success provides both a clinical and scientific foundation for this expanded therapeutic vision.

## Figures and Tables

**Table 1:** Summary of literature search strategy.

Search Element	Detail
Databases searched	PubMed, Embase, ClinicalTrials.gov
Conference sources	ASCO, ESMO, AACR, ATS annual meeting abstracts
Date range	Database inception through June 2026
Search terms	“T-cell engager”, “bispecific antibody”, “BiTE”, “thoracic malignancy”, “lung cancer”, “SCLC”, “NSCLC”, “tarlatamab”, “DLL3”, and individual target names (see Section 2 for full list)
Inclusion criteria	Peer-reviewed clinical and preclinical studies, phase 1–3 trial reports, FDA regulatory documents, systematic reviews, and conference abstracts from major oncology meetings
Exclusion criteria	Non-English publications, single case reports, and editorials without original data, unless containing mechanistic or clinical information not otherwise available
Screening approach	Single-reviewer narrative screening; formal dual-reviewer PRISMA-compliant screening and specific record counts at each stage were not tracked, consistent with the narrative review design disclosed in Section 8.2

**Table 2:** Summary of T-cell engagers under clinical evaluation in thoracic tumors.

Target/Agent	Cancer Type	Phase/Status	Key Efficacy Results	NCT #
DLL3/CD3—Tarlatamab (Imdelltra^®^)	ES-SCLC	Phase 3 completed; FDA full approval	Median OS: 13.6 vs. 8.3 mo (HR 0.60; *p* < 0.001); ORR 35% vs. 20%; Grade ≥ 3 TRAEs: 27% vs. 62% (chemo); Grade ≥ 3 all-cause AEs: 54% vs. 80%	NCT05740566
DLL3/CD3—Tarlatamab (Imdelltra^®^)	ES-SCLC	Phase 2 completed; basis for accelerated approval	ORR 40% (95% CI 31–51%; n = 99 efficacy-evaluable in 10 mg cohort); median DOR 9.7 mo; median PFS 4.9 mo; 220 total enrolled	NCT05060016
DLL3/CD3—Tarlatamab (Imdelltra^®^)	ES-SCLC + other neuroendocrine tumors	Phase 2 recruiting—UNLOCK TARLATAMAB; biomarker characterization study	Ongoing; biomarker analysis to characterize response/resistance	NCT07016230
DLL3/CD3—Tarlatamab + Sacituzumab govitecan	ES-SCLC + extrapulmonary NEC	Phase 1/2; NCI-sponsored combination trial	Ongoing; TCE + TROP-2 ADC combination	NCT07328490
DLL3/CD3—Obrixtamig (BI 764532)	SCLC/DLL3+ neuroendocrine carcinomas	Phase 1 completed; Phase 2 DAREON-5 ongoing	ORR at recommended regimens: 21% SCLC, 27% epNEC, 70% LCNEC-L; median DOR 8.5 mo; CRS 58% any grade, 2% grade ≥ 3	NCT04429087/NCT05882058
DLL3/CD3—Obrixtamig + Topotecan (DAREON-9)	Advanced ES-SCLC (2L+)	Phase 1b dose-escalation	Confirmed ORR 69% (95% CI 39–91%); unconfirmed ORR 70%; DCR 87%; n = 23 evaluable; no obrixtamig-related grade ≥ 2 neurologic events	NCT05990738
DLL3/CD3—Obrixtamig + Ezabenlimab (PD-1 inhibitor)	SCLC/DLL3+ neuroendocrine neoplasms	Phase 1 active, not recruiting—TCE + checkpoint inhibitor combination	Safety/dose-escalation ongoing	NCT05879978
DLL3/CD3/albumin—HPN328 (TriTAC)	ES-SCLC/neuroendocrine cancers	Phase 1/2 ongoing; trispecific construct with half-life extension	Confirmed ORR 50% (12/24) in SCLC and 44% (4/9) in other NEC among dose-optimization cohort patients, including one complete response in each group; CRS occurred in 59% of patients overall (30% Grade 1, 26% Grade 2, 3% Grade ≥ 3), with no Grade 3-4 CRS at target doses. DOR, PFS, and OS have not yet been reported in peer-reviewed or conference form	NCT04471727
PSMAxCD3—CC-1	Squamous cell carcinoma of the lung	Phase 1 completed	Safety/tolerability evaluation in PSMA+ squamous cell carcinoma	NCT04496674
MUC1-C/CD3—Anti-CD3-MUC1 TCE	Lung cancer	Withdrawn	Trial withdrawn; preclinical and early-phase development ongoing	NCT03501056

ORR, objective response rate; PFS, progression-free survival; OS, overall survival; DOR, duration of response; ES-SCLC, extensive-stage small cell lung cancer; SCLC, small cell lung cancer; HR, hazard ratio.

**Table 3:** CD3 T-cell engager platforms. Where specific data have not yet been reported in peer-reviewed or conference sources at the time of writing, this is noted explicitly rather than estimated.

Agent (Target)	Format/Half-Life Mechanism	Dosing	CRS: Any Grade/Grade ≥ 3	Clinical Maturity
Tarlatamab (DLL3/CD3)	Fc-containing, Fc-silenced BiTE-Fc fusion; half-life extended via FcRn-mediated recycling	Step-up IV dosing: 1 mg day 1, 10 mg day 8, then 10 mg every 2 weeks	51–61%/1%	FDA approved; phase 3 DeLLphi-304 completed
Obrixtamig (DLL3/CD3)	Fc-containing IgG-like bispecific; half-life extended via FcRn-mediated recycling	Step-up dosing per B2/B3 regimen; specific schedule not yet fully detailed in published reports	58%/2%	Phase 1 dose-escalation completed; phase 2 DAREON-5 ongoing
HPN328 (DLL3/CD3/albumin)	Fc-less TriTAC trispecific construct; half-life extended via an anti-albumin binding domain rather than an Fc domain	Dose-escalation ongoing; recommended phase 2 dose not yet established in available reports	Not yet fully reported in peer-reviewed literature at time of writing	Phase 1/2 ongoing since 2022

**Table 4:** Grade-based management of cytokine release syndrome (CRS) and immune effector cell-associated neurotoxicity syndrome (ICANS) in TCE therapy, based on ASTCT consensus grading criteria and the FDA-approved tarlatamab label.

Toxicity	Grade	Key Clinical Features	Recommended Management
CRS	Grade 1	Fever ≥ 38 °C without hypotension or hypoxia	Supportive care (antipyretics, IV fluids); continue monitoring; no dose modification typically required
CRS	Grade 2	Fever with hypotension not requiring vasopressors, and/or hypoxia responsive to low-flow oxygen	Consider tocilizumab; corticosteroids if no improvement within 24 h; hold dose until resolution to Grade ≤ 1
CRS	Grade 3–4	Hypotension requiring vasopressors, and/or hypoxia requiring high-flow oxygen or mechanical ventilation, and/or organ dysfunction	Tocilizumab plus corticosteroids; ICU-level supportive care; vasopressor and ventilatory support as needed; permanent discontinuation should be considered per label criteria
ICANS	Grade 1–2	Mild confusion, headache, attention deficits, or focal deficits without depressed consciousness	Supportive care and close neurological monitoring; dexamethasone if symptoms persist or progress; neurology consultation
ICANS	Grade 3–4	Severe confusion, depressed level of consciousness, seizures, motor weakness, or cerebral edema	High-dose dexamethasone or methylprednisolone; urgent neurology and critical care consultation; anti-seizure prophylaxis as indicated; consider anti-cytokine therapy for concurrent CRS

## Data Availability

No new data were created or analyzed in this study. Data sharing is not applicable to this article.

## Abbreviations

Abbreviations used in this manuscript are listed below:

AACR	American Association for Cancer Research
ADCC	antibody-dependent cellular cytotoxicity
ADC	antibody-drug conjugate
APC	antigen-presenting cell
ASCO	American Society of Clinical Oncology
ASTCT	American Society for Transplantation and Cellular Therapy
ATS	American Thoracic Society
BsAb	bispecific antibody
CAR	chimeric antigen receptor
CD3	cluster of differentiation 3
CD137	4-1BB, tumor necrosis factor receptor superfamily member 9
CDR	complementarity-determining region
CI	confidence interval
CEACAM5	carcinoembryonic antigen-related cell adhesion molecule5
CLDN6	claudin-6
CLDN18.2	claudin-18.2
CNS	central nervous system
CRS	cytokine release syndrome
DLL3	delta-like ligand3
DOR	duration of response
EMT	epithelial-to-mesenchymal transition
ES-SCLC	extensive-stage small cell lung cancer
FDA	U.S. Food and Drug Administration
FOLR1	folate receptor alpha
GPC3	glypican-3
HER2	human epidermal growth factor receptor 2 (ErbB2)
HR	hazard ratio
IHC	immunohistochemistry
ICI	immune checkpoint inhibitor
ICANS	immune effector cell-associated neurotoxicity syndrome
IgG	immunoglobulin G
IV	intravenous
LCNEC	large cell neuroendocrine carcinoma
LAG-3	lymphocyte-activation gene 3
MDSC	myeloid-derived suppressor cell
MHC-I	major histocompatibility complex class I
MUC1	mucin1
NCI	National Cancer Institute
NCCN	National Comprehensive Cancer Network
NEC	neuroendocrine carcinoma
NICD	Notch intracellular domain
NK	natural killer (cell)
NSCLC	non-small cell lung cancer
ORR	objective response rate
OS	overall survival
PD-1	programmed cell death protein1
PD-L1	programmed death-ligand1
PFS	progression-free survival
PSMA	prostate-specific membrane antigen
SCLC	small cell lung cancer
TAA	tumor-associated antigen
TCE	T-cell engager
TCR	T-cell receptor
TIM-3	T-cell immunoglobulin and mucin domain-3
TIL	tumor-infiltrating lymphocyte
TME	tumor microenvironment
TROP-2	trophoblast cell-surface antigen2
Treg	regulatory T cell
VISTA	V-domain Ig suppressor of T-cell activation
BiTE	bispecific T-cell engager
DART	Dual-Affinity Re-Targeting antibody
ImmTAC	Immune-mobilizing monoclonal T-cell receptor Against Cancer
TriTAC	trispecific T-cell-activating construct
STEAP1	Six-Transmembrane Epithelial Antigen of the Prostate 1
CTC	circulating tumor cell
TKI	tyrosine kinase inhibitor
EGFR	epidermal growth factor receptor
ALK	anaplastic lymphoma kinase
ROS1	ROS proto-oncogene 1
KRAS	Kirsten rat sarcoma viral proto-oncogene
ASCL1	achaete-scute homolog 1
YAP1	Yes-associated protein 1
TIGIT	T cell immunoreceptor with Ig and ITIM domains
AMPK	AMP-activated protein kinase
mTOR	mechanistic target of rapamycin
TGF-β	transforming growth factor beta
IL-6	interleukin-6
IL-10	interleukin-10
CARTOX-10	CAR T-cell therapy-associated toxicity grading tool
ICE	immune effector cell encephalopathy assessment tool
LCNEC-L	large cell neuroendocrine carcinoma of the lung
epNEC	extrapulmonary neuroendocrine carcinoma
2L+	second-line or later
RECIST	Response Evaluation Criteria in Solid Tumors
RMST	restricted mean survival time

## References

[1] Bray F., Laversanne M., Sung H., Ferlay J., Siegel R.L., Soerjomataram I., Jemal A. (2024). Global cancer statistics 2022: GLOBOCAN estimates of incidence and mortality worldwide for 36 cancers in 185 countries. CA A Cancer J. Clin..

[2] Herbst R.S., Heymach J.V., Lippman S.M. (2008). Lung cancer. N. Engl. J. Med..

[3] National Cancer Institute SEER*Explorer: An Interactive Website for SEER Cancer Statistics. Surveillance Research Program, National Cancer Institute. https://seer.cancer.gov/explorer/.

[4] Sun J.Y., Zhang D., Wu S., Xu M., Zhou X., Lu X.J., Ji J. (2020). Resistance to PD-1/PD-L1 blockade cancer immunotherapy: Mechanisms, predictive factors, and future perspectives. Biomark. Res..

[5] Zhou X., Ni Y., Liang X., Lin Y., An B., He X., Zhao X. (2022). Mechanisms of tumor resistance to immune checkpoint blockade and combination strategies to overcome resistance. Front. Immunol..

[6] Zeltsman M., Dozier J., McGee E., Ngai D., Adusumilli P.S. (2017). CAR T-cell therapy for lung cancer and malignant pleural mesothelioma. Transl. Res..

[7] Chocarro L., Arasanz H., Fernandez-Rubio L., Blanco E., Echaide M., Bocanegra A., Teijeira L., Garnica M., Morilla I., Martinez-Aguillo M. (2022). CAR-T Cells for the Treatment of Lung Cancer. Life.

[8] Zhang Q., Fu Q., Hou Y., Lv Y., Cao C., Li X. (2025). Advances in CAR-T therapy for small cell lung cancer. Int. J. Cancer.

[9] Byers L.A., Sable B., Smit M.D., Dowlati A., Spigel D.R., Steuer C.E., Owonikoko T.K., Tripathy D., Johnson M.L., Paz-Ares L.G. (2019). Phase 1 study of AMG 119, a chimeric antigen receptor (CAR) T cell therapy targeting DLL3, in patients with relapsed/refractory small cell lung cancer (SCLC). J. Clin. Oncol..

[10] Chen T., Wang M., Chen Y., Liu Y. (2024). Current challenges and therapeutic advances of CAR-T cell therapy for solid tumors. Cancer Cell Int..

[11] Morgan R.A., Yang J.C., Kitano M., Dudley M.E., Laurencot C.M., Rosenberg S.A. (2010). Case report of a serious adverse event following the administration of T cells transduced with a chimeric antigen receptor recognizing ERBB2. Mol. Ther..

[12] Lotze M.T., Maeurer M., Quezada S.A., Coukos G. (2024). Lung Cancer Adoptive Cell Therapy: Inspiring TIL ACT Comes Center Stage. Cancer Discov..

[13] Baulu E., Gardet C., Chuvin N., Depil S. (2023). TCR-engineered T cell therapy in solid tumors: State of the art and perspectives. Sci. Adv..

[14] Malviya M., Aretz Z.E.H., Molvi Z., Farabella I., Mesecke S., D’Amico S., Chiti S., Passaro A., de Braud F., Garassino M.C. (2023). Challenges and solutions for therapeutic TCR-based agents. Immunol. Rev..

[15] Shanshal M., Caimi P.F., Adjei A.A., Ma W.W. (2023). T-Cell Engagers in Solid Cancers-Current Landscape and Future Directions. Cancers.

[16] Ahn M.J., Cho B.C., Felip E., Korantzonis J., Ohashi K., Majem M., Dowlati A., Chiang A.C., Thomas M., Greystoke A. (2023). Tarlatamab for Patients with Previously Treated Small-Cell Lung Cancer. N. Engl. J. Med..

[17] Mountzios G., Sun L., Cho B.C., Demirci U., Baka S., Gümüş M., Lugini A., Zhu B., Yu Y., Korantzis I. (2025). Tarlatamab in small-cell lung cancer after platinum-based chemotherapy. N. Engl. J. Med..

[18] Goebeler M.E., Stuhler G., Bargou R. (2024). Bispecific and multispecific antibodies in oncology: Opportunities and challenges. Nat. Rev. Clin. Oncol..

[19] Li H., Er Saw P., Song E. (2020). Challenges and strategies for next-generation bispecific antibody-based antitumor therapeutics. Cell. Mol. Immunol..

[20] Haber L., Olson K., Kelly M.P., Crawford A., DiLillo D.J., Smith E.J., Jalal S., Tschantz W.R., Chen S., Deng L. (2021). Generation of T-cell-redirecting bispecific antibodies with differentiated profiles of cytokine release and biodistribution by CD3 affinity tuning. Sci. Rep..

[21] Abdelmotaleb O., Schneider A., Waldhauer I., Ast O., Sam J., Freimoser-Grundschober A., Moessner E., Klein C., Bacac M., Umansky V. (2025). Optimizing efficacy and safety of T cell bispecific antibodies: The interdependence of CD3 and tumor antigen binder affinities in FOLR1 and CEACAM5 2+1 TCBs. mAbs.

[22] Wu D., Huang L., Naren G., Liu J., Wang Y., Zhao C., Sun L., Chen Y., Xu X., Zhang H. (2025). Optimization of a novel 2+2 BCMA x CD3 bispecific antibody for minimized cytokine release and potent efficacy. Mol. Cancer Ther..

[23] Santich B.H., Park J.A., Tran H., Guo H.F., Huse M., Cheung N.V. (2020). Interdomain spacing and spatial configuration drive the potency of IgG-[L]-scFv T cell bispecific antibodies. Sci. Transl. Med..

[24] Morris E.C., Neelapu S.S., Giavridis T., Sadelain M. (2022). Cytokine release syndrome and associated neurotoxicity in cancer immunotherapy. Nat. Rev. Immunol..

[25] Kiefer M., Feige M.J., Skerra A., Lehmann A., Weber C., Hoffmann F., Schmidt S., Fischer D., Becker K., Bauer M. (2024). SAIL66, a next generation CLDN6-targeting T-cell engager, demonstrates potent antitumor efficacy through dual binding to CD3/CD137. J. Immunother. Cancer.

[26] Sergeeva O.A., Jin G., Sarkar M., Xu R., Zhang P., Wang H., Liu Y., Chen X., Zhao Y., Li J. (2025). EVOLVE platform, a trispecific T cell engager with integrated CD2 costimulation, for the treatment of solid and hematologic tumors. Proc. Natl. Acad. Sci. USA.

[27] Kim N., Kwon I. (2026). Conditionally activatable antibody platforms: Mechanisms, modalities, and clinical translation potential. BioDrugs.

[28] Panchal A., Seto P., Wall R., Williams S., Liu X., Zhang Y., Tan Z., Chen G., Li M., Zhou Y. (2020). COBRA: A highly potent conditionally active T cell engager engineered for the treatment of solid tumors. mAbs.

[29] Barone A., Shah R., Sickafuse S., Zhang L., Zhao H., Lemery S.J., Beaver J.A., Pazdur R., Singh H., Blumenthal G.M. (2026). FDA Approval Summary: Tarlatamab for the treatment of extensive stage small cell lung cancer. Clin. Cancer Res..

[30] US Food and Drug Administration FDA Grants Traditional Approval to Tarlatamab-Dlle for Extensive Stage Small Cell Lung Cancer. https://www.fda.gov/drugs/resources-information-approved-drugs/fda-grants-traditional-approval-tarlatamab-dlle-extensive-stage-small-cell-lung-cancer.

[31] Rudin C., Mountzios G., Sun L., Cho B.C., Felip E., Ahn M.J., Dowlati A., Spigel D.R., Morgensztern D., Johnson M.L. (2025). Tarlatamab versus chemotherapy as second-line treatment for small cell lung cancer (SCLC): Primary analysis of Ph3 DeLLphi-304. J. Clin. Oncol..

[32] Aix S.P., Ciuleanu T.E., Navarro A., Biesma B., Noppen C., Heigener D., Levchenko E., Garcia-Campelo R., Grohe C., Felip E. (2023). Combination lurbinectedin and doxorubicin versus physician’s choice of chemotherapy in patients with relapsed small-cell lung cancer (ATLANTIS): A multicentre, randomised, open-label, phase 3 trial. Lancet Respir. Med..

[33] Jazz Pharmaceuticals Jazz Pharmaceuticals Provides Update on Zepzelca (Lurbinectedin) Phase 3 LAGOON Trial in Second-Line Small Cell Lung Cancer. Press Release, 12 June 2026. https://www.prnewswire.com/news-releases/jazz-pharmaceuticals-provides-update-on-zepzelca-lurbinectedin-phase-3-lagoon-trial-in-second-line-small-cell-lung-cancer-302798651.html.

[34] Wang J., Takundwa R., Yang H., Chen X., Liu Y., Zhang Z., Li W., Zhao Y., Xu R., Wang L. (2026). Comparative efficacy of tarlatamab and second-line therapies in extensive-stage small cell lung cancer: A network meta-analysis. Lung Cancer.

[35] Ganti A.P., Loo B.W., Badiyan S., Bassetti M., Bestvina C., Chiang A., Choudhury N., D’Avella C.A., Daly M., Dowlati A. (2026). NCCN Guidelines Insights: Small Cell Lung Cancer, Version 2.2026. J. Natl. Compr. Cancer Netw..

[36] Mountzios G.S., Cho B.C., Lammers P., Sun L., Arulananda S., Blackhall F.H., Yoshida T., Ahn M.J., Punekar S.R., Zhu B. (2026). Intracranial efficacy of tarlatamab versus chemotherapy (CTx) as second-line (2L) treatment for small cell lung cancer (SCLC): DeLLphi-304 phase 3 post hoc analysis. J. Clin. Oncol..

[37] Paulson K.G., Lau S.C.M., Ahn M.J., Moskovitz M., Pogorzelski M., Hafliger S., Parkes A., Zhang Y., Hamidi A., Thompson C.G. (2025). Safety and activity of tarlatamab in combination with a PD-L1 inhibitor as first-line maintenance therapy after chemo-immunotherapy in patients with extensive-stage small-cell lung cancer (DeLLphi-303): A multicentre, non-randomised, phase 1b study. Lancet Oncol..

[38] Wermke M., Gambardella V., Kuboki Y., Felip E., Sanmamed M.F., Alese O.B., Sayehli C.M., Arriola E., Wolf J., Villaruz L.C. (2025). Phase I dose-escalation results for the delta-like ligand 3/CD3 IgG-like T-cell engager obrixtamig (BI 764532) in patients with delta-like ligand 3+ small cell lung cancer or neuroendocrine carcinomas. J. Clin. Oncol..

[39] Wermke M., Alt J., Bozorgmehr F., Fuchs F., du Rusquec P., Gazzah A., Dulloo S., Kalinka E., Chauhan A., Mosikian A. (2025). DAREON-9, a phase Ib study of obrixtamig plus topotecan in patients with advanced small cell lung cancer (SCLC): Interim analysis results. J. Clin. Oncol..

[40] Hassel J.C., Piperno-Neumann S., Rutkowski P., Baurain J.F., Schlaak M., Butler M.O., Sullivan R.J., Dummer R., Kirkwood J.M., Joshua A.M. (2023). Three-year overall survival with tebentafusp in metastatic uveal melanoma. N. Engl. J. Med..

[41] Kelly W.K., Danila D.C., Lin C.C., Lee J.L., Matsubara N., Ward P.J., Armstrong A.J., Pook D., Kim M., Dorff T.B. (2024). Xaluritamig, a STEAP1 x CD3 XmAb 2+1 immune therapy for metastatic castration-resistant prostate cancer. Cancer Discov..

[42] Ma Z., Zhou Z., Duan W., Zhang Y., Wang X., Li J., Liu Y., Chen H., Xu L., Zhao M. (2024). DR30318, a novel tri-specific T cell engager for Claudin 18.2 positive cancers immunotherapy. Cancer Immunol. Immunother..

[43] De Sanctis F., Dusi S., Caligola S., Canè S., Trovato R., Ugel S., De Cicco P., Fiore A., Sartoris S., Solito S. (2024). Expression of the membrane tetraspanin claudin 18 on cancer cells promotes T lymphocyte infiltration and antitumor immunity in pancreatic cancer. Immunity.

[44] Skoulidis F., Heymach J.V. (2019). Co-occurring genomic alterations in non-small-cell lung cancer biology and therapy. Nat. Rev. Cancer.

[45] Hsu Y.H.R., Almutrafi A., Hueniken K., Tsao M.S., Liu G., Leighl N.B., Shepherd F.A., Feld R., Bradbury P.A., Chen Z. (2025). The landscape of CEACAM5 expression by immunohistochemistry in NSCLC. JTO Clin. Res. Rep..

[46] Segal N.H., Melero I., Moreno V., Boni V., Calvo E., Rodriguez-Ruiz M.E., Perez-Gracia J.L., Alonso G., Patel M.R., Powderly J. (2024). CEA-CD3 bispecific antibody cibisatamab with or without atezolizumab in patients with CEA-positive solid tumours: Results of two multi-institutional Phase 1 trials. Nat. Commun..

[47] Qiu S., Zhang J., Wang Z., Chen X., Liu Y., Xu R., Li W., Zhao Y., Wang L., Zhang Y. (2023). Targeting Trop-2 in cancer: Recent research progress and clinical application. Biochim. Biophys. Acta-Rev. Cancer.

[48] Guerra E., Alberti S. (2022). The anti-Trop-2 antibody-drug conjugate Sacituzumab Govitecan-effectiveness, pitfalls and promises. Ann. Transl. Med..

[49] Wang X., Long M., Dong K., Zhang Y., Liu Y., Chen X., Li W., Zhao Y., Xu R., Wang L. (2013). Chemotherapy agents-induced immunoresistance in lung cancer cells could be reversed by trop-2 inhibition in vitro and in vivo by interaction with MAPK signaling pathway. Cancer Biol. Ther..

[50] Li B.T., Smit E.F., Goto Y., Nakagawa K., Udagawa H., Mazières J., Nagasaka M., Bazhenova L., Saltos A.N., Felip E. (2022). Trastuzumab deruxtecan in HER2-mutant non-small-cell lung cancer. N. Engl. J. Med..

[51] Cheung A., Bax H.J., Josephs D.H., Ilieva K.M., Bracher M., Spiliotamaki G., Crescioli S., Walton I., Karagiannis P., Dodev T.S. (2016). Targeting folate receptor alpha for cancer treatment. Oncotarget.

[52] Call J.A., Orr D.W., Anderson I.C., Hamilton E.P., Moore K.N., Richardson D.L., Ulahannan S.V., Spira A.I., Patel M.R., Conkling P.R. (2023). Phase 1/2 study of PRO1184, a novel folate receptor alpha-directed antibody-drug conjugate, in patients with locally advanced and/or metastatic solid tumors. J. Clin. Oncol..

[53] Flieswasser T., Van den Eynde A., Freire Boullosa L., De Waele J., Van Audenaerde J.R., Marcq E., Lardon F., Smits E.L., Pauwels P., Jacobs J. (2023). Targeting CD70 in combination with chemotherapy to enhance the anti-tumor immune effects in non-small cell lung cancer. OncoImmunology.

[54] Jacobs J., Zwaenepoel K., Rolfo C., Van den Bossche J., Deben C., Siozopoulou V., Santermans E., Deschoolmeester V., Janssens A., Lardon F. (2015). Unlocking the potential of CD70 as a novel immunotherapeutic target for non-small cell lung cancer. Oncotarget.

[55] Ning J., Jiang S., Li X., Zhang Y., Liu Y., Chen X., Zhao Y., Xu R., Wang L., Li W. (2021). GPC3 affects the prognosis of lung adenocarcinoma and lung squamous cell carcinoma. BMC Pulm. Med..

[56] Nath S., Mukherjee P. (2014). MUC1: A multifaceted oncoprotein with a key role in cancer progression. Trends Mol. Med..

[57] Jasuja R., Raina D., Ahmad R., Kufe D., Zhang Y., Liu Y., Chen X., Zhao Y., Xu R., Wang L. (2024). Evaluation of MUC1-C/CD3 biparatopic-bispecific (BPBS) T cell engager as an immunotherapeutic agent for the treatment of MUC1-expressing metastatic breast cancer (mBC). Ann. Oncol..

[58] Stadler C.R., Bähr-Mahmud H., Plum L.M., Schmoldt K., Kölsch A.C., Türeci Ö., Sahin U. (2016). Characterization of the first-in-class T-cell-engaging bispecific single-chain antibody for targeted immunotherapy of solid tumors expressing the oncofetal protein claudin 6. OncoImmunology.

[59] Li M., Che N., Feng Y., Zhang Y., Liu Y., Chen X., Zhao Y., Xu R., Wang L., Li W. (2022). B7-H4 expression promotes non-small cell lung cancer progression via AMPK/mTOR signaling. Exp. Mol. Pathol..

[60] Hackenbruch C., Heitmann J.S., Walz J.S., Zender L., Lauer U.M., Bitzer M., Malek N.P., Schmidt M., Tabatabai G., Thorwarth M. (2023). Clinical development of the bispecific PSMAxCD3 antibody CC-1. J. Clin. Oncol..

[61] Wang H.L., Wang S.S., Song W.H., Pan Y., Yu L., Sillanpää A., Kuruma H., Zhang Y., Liu Y., Chen X. (2015). Expression of Prostate-Specific Membrane Antigen in Lung Cancer Cells and Tumor Neovasculature Endothelial Cells and Its Clinical Significance. PLoS ONE.

[62] Mishra A., Meador C.B., Kikkeri K., Cunneely Q., Lin M., Carmona-LaSalle T.J., Huang S.B., Bell R., Putaturo V., Xia W. (2026). Circulating tumor cells predict response to the DLL3-targeting bispecific antibody tarlatamab. Cancer Discov..

[63] Gerard A., Hueso T., Laparra A., Bihan K., Bihan C., Lebrun-Vignes B., Salem J.E., Funck-Brentano E., Moslehi J.J., Johnson D.B. (2024). Reactions and adverse events induced by T-cell engagers as anti-cancer immunotherapies, a comprehensive review. Eur. J. Cancer.

[64] Shimabukuro-Vornhagen A., Godel P., Subklewe M., Stemmler H.J., Schlößer H.A., Schlaak M., Kochanek M., Böll B., von Bergwelt-Baildon M.S. (2018). Cytokine release syndrome. J. Immunother. Cancer.

[65] Lee D.W., Santomasso B.D., Locke F.L., Ghobadi A., Turtle C.J., Brudno J.N., Maus M.V., Park J.H., Litzow M.R., Kalos M. (2019). ASTCT Consensus Grading for Cytokine Release Syndrome and Neurologic Toxicity Associated with Immune Effector Cells. Biol. Blood Marrow Transplant..

[66] Santomasso B.D., Nastoupil L.J., Adkins S., Lacchetti C., Schneider B.J., Anadkat M.J., Atkins M.B., Barker C.A., Daniels G.A., Fecher L.A. (2021). Management of Immune-Related Adverse Events in Patients Treated with Chimeric Antigen Receptor T-Cell Therapy: ASCO Guideline. J. Clin. Oncol..

[67] Blocker S., Kim D., Cavalieri C.C., Cannon S., Cass A., Olinger A., Bortka B., Gustafson B., Schepers A., Hobbs J. (2026). Risk factors for CRS and ICANS with tarlatamab in small cell lung cancer and associated efficacy outcomes: Insights from clinical practice. Eur. J. Cancer.

[68] Xiao Z., Todd L., Huang L., Lu Z., Wang Y., Zhang H., Sun L., Chen Y., Xu X., Zhang H. (2023). Desmoplastic stroma restricts T cell extravasation and mediates immune exclusion and immunosuppression in solid tumors. Nat. Commun..

[69] Ellerman D. (2019). Bispecific T-cell engagers: Towards understanding variables influencing the in vitro potency and tumor selectivity and their modulation to enhance their efficacy and safety. Methods.

[70] Arenas E.J., Martínez-Sabadell A., Rius Ruiz I., Román Alonso M., Escorihuela M., Luque A., Fajardo C.A., Gros A., Klein C., Arribas J. (2021). Acquired cancer cell resistance to T cell bispecific antibodies and CAR T targeting HER2 through JAK2 down-modulation. Nat. Commun..

[71] O’Donnell J.S., Teng M.W.L., Smyth M.J. (2019). Cancer immunoediting and resistance to T cell-based immunotherapy. Nat. Rev. Clin. Oncol..

